# Dietary phosphorus consumption alters T cell populations, cytokine production, and bone volume in mice

**DOI:** 10.1172/jci.insight.154729

**Published:** 2023-05-22

**Authors:** Joseph L. Roberts, Mingcan Yu, Manjula Viggeswarapu, Jamie L. Arnst, Roberto Pacifici, George R. Beck

**Affiliations:** 1 The Atlanta Department of Veterans Affairs Medical Center, Decatur, Georgia, USA.; 2 Nutrition and Health Sciences, Laney Graduate School, Emory University, Atlanta, Georgia, USA.; 3 Emory University, Department of Medicine, Division of Endocrinology, Metabolism and Lipids, Atlanta, Georgia, USA.; 4 Department of Medicine, Division of Endocrinology, Metabolism and Lipids, Immunology and Molecular Pathogenesis Program, Emory Microbiome Research Center, Emory University, Atlanta, Georgia, USA.; 5 The Winship Cancer Institute, Emory University School of Medicine, Atlanta Georgia, USA.

**Keywords:** Bone Biology, Osteoporosis

## Abstract

The intake of dietary phosphate far exceeds recommended levels; however, the long-term health consequences remain relatively unknown. Here, the chronic physiological response to sustained elevated and reduced dietary phosphate consumption was investigated in mice. Although serum phosphate levels were brought into homeostatic balance, the prolonged intake of a high-phosphate diet dramatically and negatively impacted bone volume; generated a sustained increase in the phosphate responsive circulating factors FGF23, PTH, osteopontin and osteocalcin; and produced a chronic low-grade inflammatory state in the BM, marked by increased numbers of T cells expressing IL-17a, RANKL, and TNF-α. In contrast, a low-phosphate diet preserved trabecular bone while increasing cortical bone volume over time, and it reduced inflammatory T cell populations. Cell-based studies identified a direct response of T cells to elevated extracellular phosphate. Neutralizing antibodies against proosteoclastic cytokines RANKL, TNF-α, and IL-17a blunted the high-phosphate diet–induced bone loss identifying bone resorption as a regulatory mechanism. Collectively, this study illuminates that habitual consumption of a high-phosphate diet in mice induces chronic inflammation in bone, even in the absence of elevated serum phosphate. Furthermore, the study supports the concept that a reduced phosphate diet may be a simple yet effective strategy to reduce inflammation and improve bone health during aging.

## Introduction

There is a growing appreciation that changes in serum phosphorus/phosphate (Pi) levels, even in the context of normal renal function, influence age-associated disease progression as observed with cancer (1–4), bone metabolism (5–10), and renal and cardiovascular function (11–13). Changes in serum Pi levels are generally proportional to dietary intake in healthy adults. High dietary Pi intake is particularly relevant for individuals in the United States, as population surveys have demonstrated that the average Pi intake for adults in the United States far exceeds the recommended intake by the Food and Drug Administration/Institute of Medicine (FDA/IOM) (700 mg/d) (14), with those in the ninetieth percentile exceeding 2,500–3,000 mg/d (9, 15). This is thought to be partly due to frequent consumption of convenience foods, which have been estimated to add more than 1,000 mg/d of Pi to daily consumption (16). Additionally, these epidemiological studies likely underestimate the true intake of Pi due to the fact that nutrient composition databases do not fully account for Pi as a food additive (5, 15, 17). Compounding the high overall daily intake, Pi from highly processed foods is often in the form of inorganic salts, which are more efficiently absorbed (80%–100%) than naturally occurring phosphates (often in the form of phytate) that can require enzymatic digestion (40%–60% absorbed) (5, 18). Although it has been suggested that high dietary Pi consumption accelerates mammalian aging (19), currently, the long-term consequences of a sustained diet high in Pi are only minimally understood, while the possible health benefits of a reduced Pi diet are essentially unknown.

The importance of Pi homeostasis on long-term health is exemplified by KO or mutation of phosphaturic factors such as the osteocyte-derived hormone fibroblast growth factor 23 (FGF23) or its receptor binding cofactor Klotho, and this results in hyperphosphatemia, inflammation, and a premature-aging syndrome in mice (20, 21). Interestingly, these pathologies can be mostly corrected with a low-Pi diet (LPD) (19), providing proof of principal in the power of modulating Pi consumption for health benefits. Systemic changes in serum Pi that occur from high dietary Pi consumption are countered by a coordinated network of circulating endocrine factors, the dominant being FGF23 and parathyroid hormone (PTH). FGF23 and PTH act to decrease serum Pi primarily by reducing Pi reabsorption in the kidney, which in turn increases urinary Pi excretion (22–24). There is also a growing list of factors that are responsive to changes in serum and dietary consumption of Pi but whose function in Pi homeostasis remains to be fully elucidated such as osteopontin (OPN). Furthermore, it is becoming increasingly clear that Pi-responsive factors are potent biological agents that have Pi-independent effects on various tissues, and these factors have been implicated in the development of diseases affecting numerous tissues, including those of the skeletal, cardiovascular, renal, and immune systems (25). Therefore, a better understanding of relationship to Pi homeostasis and long-term health is needed.

Pi and calcium (Ca) are the main constituents of bone mineral; accordingly, it is logical that the skeleton is responsive to changes in Pi. However, paradoxically, increased serum Pi has been linked to a decrease in bone density (6, 7, 26–28). Bone health in adults is maintained through the continuous process of remodeling in which old bone is resorbed by osteoclasts and in which the mineral is replaced by bone forming osteoblasts. The formation of osteoclasts is stimulated by receptor activator of NF-κB ligand (RANKL), which is produced by osteoblast lineage and cells of the immune system (29), binding to RANK on preosteoclasts. The goals of this study were, therefore, to understand the temporal systemic effects of chronic high and low Pi consumption on bone metabolism and to gain insight into the underlying mechanisms driving these effects. A detailed longitudinal study was performed in adult female mice fed LPD (0.2% Pi), normal-Pi diet (NPD, 0.6% Pi), or high-Pi diet (HPD, 1.8% Pi) for up to 20 weeks. Results identify chronic systemic effects and health consequences of habitually high and reduced dietary Pi consumption, including a strong and sustained influence on both trabecular and cortical bone; a persistent increase in FGF23, PTH, OPN, and osteocalcin in the context of unremarkable serum Pi and Ca; identification of the requirement of RANKL and TNF-α signaling for dietary Pi–induced bone loss; the direct effect of elevated Pi on T cell expression of RANKL, TNF-α, and IL-17a; and a potentially novel link between sustained high dietary Pi consumption and inflammation.

## Results

### Long-term dietary Pi consumption levels alter serum mineral levels but not weight.

To better understand the long-term effects of the common dietary element Pi on health and disease, a longitudinal study was performed with 3 diets with varying Pi content: (a) LPD (0.2% Pi content), (b) NPD (0.6% Pi content), and (c) HPD (1.8% Pi content) with constant Ca (0.6%) and 2.2 IU vitamin D, thereby creating Pi/Ca ratios of 1:3, 1:1, and 3:1. These ratios generally represent the top and bottom fifth to tenth percentiles of Pi consumption according to recent National Health and Nutrition Examination Survey (NHANES) data (14, 30). The diets were designed to be isocaloric with similar calories by fat and total energy (Supplemental Table 1; supplemental material available online with this article; https://doi.org/10.1172/jci.insight.154729DS1). Female C57BL/6J mice at 10 weeks of age were randomly assigned to 1 of the 3 diets for 0 hours (baseline), 6 hours, or 1, 2.5, 5, 10, or 20 weeks. Mice were weighed at the completion of the time points (sacrifice), and no effect of diet on body weight was found, suggesting that no gross toxicity associated with long-term consumption of either the HPD or LPD (Supplemental Figure 1). Phosphate, Ca, and iron levels were measured in serum from the baseline (0-hour), 6-hour, 1-week, and 20-week mice. A strong correlation between Pi consumption and serum levels was found early in the response but ultimately returned close to a homeostatic balance by 20 weeks (Table 1). Serum Ca was lower in the HPD-fed group at 1 week relative to NPD, which was eventually normalized by 20 weeks. These results are generally in line with reports from humans, albeit at shorter time frames (16, 31–34). Iron demonstrated the strongest relationship to dietary Pi intake over time, with the low Pi–consuming mice exhibiting higher serum iron levels while HPD resulted in a decrease at 1 and 20 weeks (Table 1).

### Dietary Pi alters trabecular microarchitecture.

**Figure 1:**
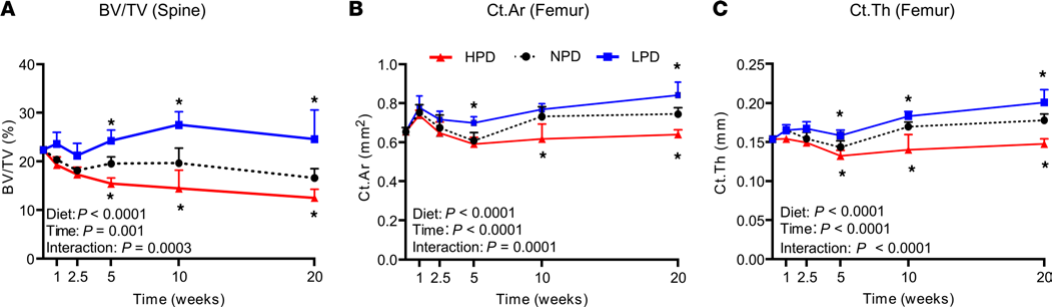
Impact of phosphorus consumption on vertebral trabecular and femoral cortical volume over time. Female C57BL/6J mice (10 weeks of age) were randomized to receive NPD, LPD, or HPD for 1, 2.5, 5, 10, or 20 weeks, and trabecular and cortical indices were quantified by μCT and plotted against time. (**A**) Vertebral bone volume/tissue volume (BV/TV). (**B**) Femoral cortical area (Ct.Ar). (**C**) Femoral cortical thickness (Ct.Th). An ordinary 2-way ANOVA determined diet effect, time effect, and time by diet interaction effect. To determine differences between LPD and HPD compared with the NPD control diet at each time point, the Dunnett’s multiple-comparison test was used. *P < 0.05 relative to NPD by Student’s *t* test. Data represent mean ± SD. NPD, normal-phosphate diet; LPD, low-phosphate diet; HPD, high-phosphate diet. *n* = 5–10/group.

High dietary Pi has been reported to negatively influence the skeleton (7, 26, 27, 35–38); however, this relationship relied on cross-sectional data at single time points. Thus, to determine the longitudinal effects of both high and low Pi consumption on bone volume, spine trabecular microarchitectural indices were assessed by ex vivo μCT as a function of time. Representative 3D images of spine trabecular microarchitecture visually illustrate changes in bone across diets and over time (Supplemental Figure 2). There was a significant effect of diet and time for most trabecular indices, including bone volume/tissue volume (BV/TV) (Figure 1A), trabecular number (Tb.N), trabecular thickness (Tb.Th), and trabecular spacing (Tb.Sp) (Supplemental Table 2 and Supplemental Figure 3). Furthermore, there was a significant interaction between diet and time for all trabecular morphological parameters. Comparison of HPD or LPD relative to NPD at each time point identified robust and significant differences from NPD by 5 weeks. Other indices (Tb.N, Tb.Th, and Tb.Sp) followed a similar pattern as would be expected (Supplemental Figure 3). The increase in the spine BV/TV with LPD correlated with an increase in Tb.Th as opposed to number, whereas the bone loss associated with HPD corresponded more closely to a decrease in Tb.N. Together, the results identify compartment-specific beneficial effects of a LPD on trabecular bone, whereas HPD resulted in sustained deterioration in bone microstructure.

### Effects of dietary Pi on cortical bone.

The influence of varying dietary Pi on changes in cortical indices over time was also assessed by μCT of the femur, an important indication of bone strength and fracture risk. Representative images of femur cortical structure illustrate changes in bone microarchitecture across diets and over time (Supplemental Figure 4). There was an increase in cortical area (Ct.Ar) and cortical thickness (Ct.Th) over time in all groups; however, the LPD and NPD groups increased substantially more than HPD, which remained mostly unchanged over time (Figure 1, B and C). There was a highly significant diet, time, and diet-time interactions for Ct.Ar and Ct.Th microarchitecture. Similar to trabecular indices, significant differences in Ct.Ar and Ct.Th became apparent between HPD and LPD by 5 weeks (Figure 1, B and C, and Supplemental Table 3). In contrast to trabecular indices, time had a positive effect on cortical bone from mice fed a NPD. However, mice fed a HPD remained essentially unchanged, suggesting an inhibition of age-related increases in cortical structure.

### Effects of dietary Pi consumption on serum makers of bone metabolism.

**Figure 2:**
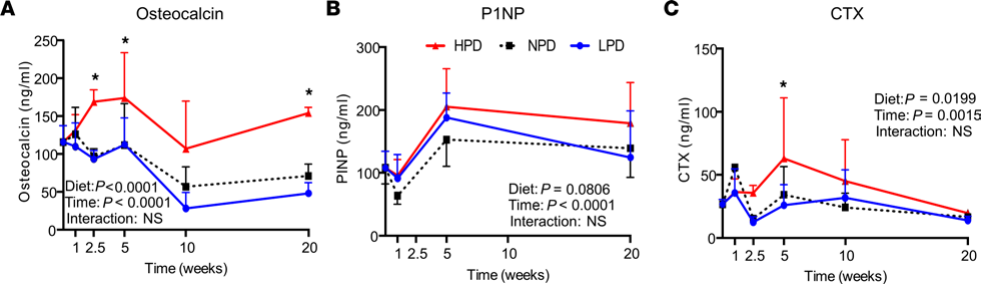
Effect of phosphorus consumption and time on serum markers of bone metabolism. (**A**–**C**) Serum from the mice described in Figure 1 was assessed for levels of bone metabolism markers by ELISA: osteocalcin, P1NP, and CTX. An ordinary 2-way ANOVA determined diet effect, time effect, andtime by diet interaction effect. To determine differences between LPD and HPD compared with the NPD control diet at each time point, the Dunnett’s multiple-comparison test was used. *P < 0.05 relative to NPD by Student’s *t* test. Data represent mean ± SD. *n* = 5–10/group.

Temporal changes of serum markers of bone metabolism were also assessed. Bone formation is commonly assessed by increases in osteocalcin and procollagen type 1 N-terminal propeptide (P1NP), whereas bone resorption is reflected by Carboxy-terminal telopeptide of type 1 collagen (CTX). A significant effect of time was found for all 3 markers measured (Figure 2, A–C). Only CTX and osteocalcin demonstrated a significant effect of diet, and no significant interaction between diet and time was observed for any factor (Figure 2, A–C, and Supplemental Table 4). There was an initial elevation of CTX in HPD-fed mice through 5 weeks, but the levels declined over time to levels similar to NPD and LPD, suggesting an initial increase in bone resorption in response to HPD. HPD resulted in increased serum osteocalcin starting at 2.5 weeks compared with NPD, which remained elevated throughout the 20-week study period. Although not statistically significant, P1NP was modestly elevated in both HPD and LPD relative to NPD early in the response. Together, the results suggest that LPD drives a small increase in formation in the absence of increased resorption, and this is sufficient to increase bone volume; however, HPD drives a significant increase in resorption that outpaces the small increase in formation, resulting in bone loss. Unexpectedly, osteocalcin a traditional marker of formation, was strongly elevated throughout the time course in response to HPD. Osteocalcin is known to have functions beyond bone formation, and the result identifies a potentially novel role of osteocalcin in phosphate homeostasis.

### Pi consumption regulates phosphate-responsive mineral metabolism endocrine factors.

**Figure 3:**
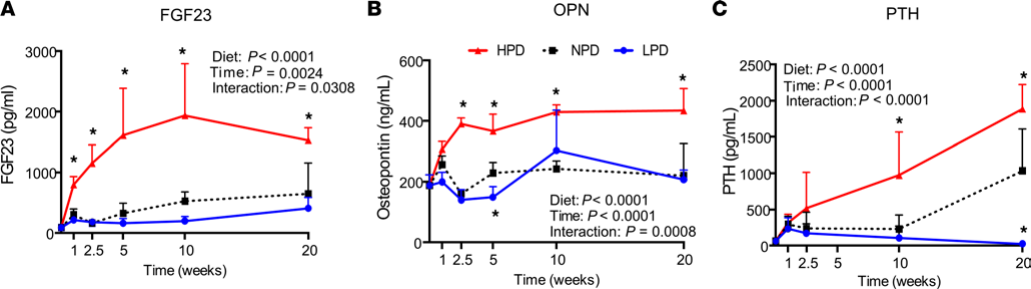
Effect of phosphorus consumption and time on phosphorus-responsive endocrine factors. (**A**–**C**) Serum from the mice described in Figure 1 was assessed for levels of phosphate responsive endocrine factors by ELISA: FGF23, osteopontin, and PTH. An ordinary 2-way ANOVA determined diet effect, time effect, and time by diet interaction effect. To determine differences between LPD and HPD compared with the NPD control diet at each time point, the Dunnett’s multiple-comparison test was used. *P < 0.05 relative to NPD by Student’s *t* test. Data represent mean ± SD. *n* = 5–10/group.

Serum was also analyzed for the Pi-responsive circulating mineral metabolism factors FGF23, PTH, and OPN. The effect of diet and time, and an interaction between diet and time, was significant for all serum factors measured (Figure 3, A–C, and Supplemental Table 5). Compared with NPD, HPD stimulated an increase in serum FGF23, OPN, and PTH, with serum FGF23 having the earliest sustained increase. The FGF23 ELISA measured the C-terminal FGF23, which recognizes both cleaved and biologically active intact FGF23; thus, an ELISA that specifically measures intact FGF23 was utilized that revealed a similar response (Supplemental Figure 5). The strong correlation identifies that the majority of circulating FGF23 throughout the time course is the intact form. In response to LPD (relative to NPD), there was an early decrease in serum OPN, whereas FGF23 was only moderately decreased, with both factors generally returning to levels comparable with NPD control mice by 20 weeks. LPD decreased PTH relative to NPD only at 20 weeks. The results identify the long-term elevation of these mineral metabolism endocrine factors in response to a sustained HPD, despite unremarkable serum Pi levels.

### HPD-induced bone loss is blunted in immune-compromised mice.

**Figure 4:**
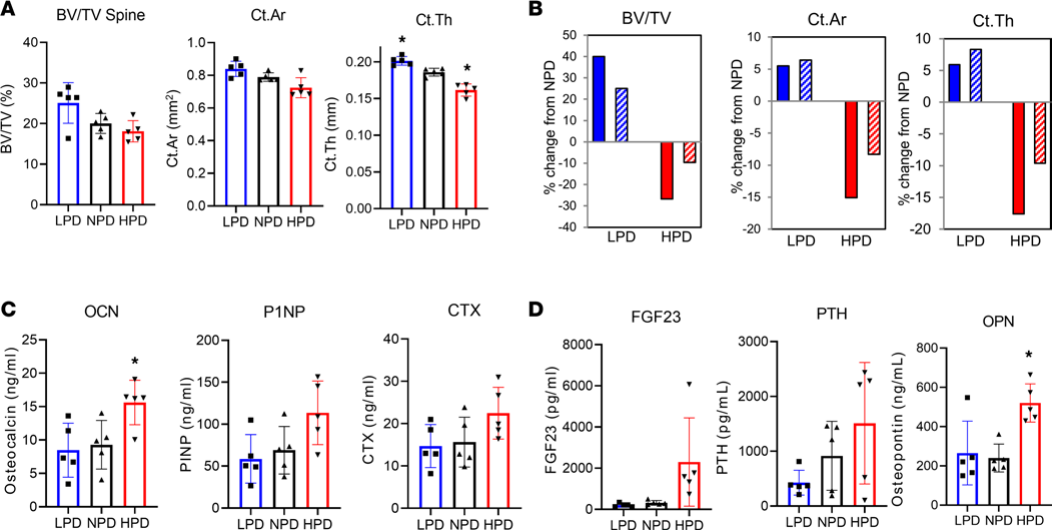
High-Pi–induced bone loss is blunted in immunocompromised mice. Ten-week-old female RAG2-KO mice were fed LPD, NPD, or HPD for 10 weeks, and serum and bones were collected. (**A**) μCT was used to quantitate trabecular and cortical volume of spine. (**B**) Presented as percent change from NPD for control (data from Figure 1; solid bars) and RAG2^–/–^ mice (striped bars). (**C** and **D**) Serum was analyzed for bone metabolism markers (**C**) and endocrine factors (**D**) by ELISA. *n* = 5. Data are shown as mean ± SD. *P < 0.05 relative to NPD by Student’s *t* test.

Based on the associative relationships between high serum Pi, aging, and multiple disease pathologies, including bone loss, we hypothesized that a HPD might influence inflammation, representing a potentially novel mechanistic link. To investigate the functional requirement of the immune system for HPD-induced bone loss, 10-week-old female Rag2^–/–^ mice were fed LPD, NPD, or HPD for 10 weeks. The Rag2 mutation blocks maturation of T and B cells because of the inability to initiate V(D)J rearrangement and, without challenge, are generally healthy (39). Weights were similar between groups, suggesting no gross health effects of the diets (not shown). Analysis of spine and femur by μCT demonstrated that the loss of trabecular and cortical bone volume in response to HPD was substantially blunted in RAG2 mice fed HPD for 10 weeks relative to control; however, the bone gain in response to LPD was similar to WT controls (Figure 4, A and B). No differences were identified in CTX or P1NP between diets, similar to WT mice at this time (Figure 2). However, an increase in osteocalcin was identified in the RAG2^–/–^ mice fed a HPD (Figure 4C) as well as in the Pi-responsive endocrine factor OPN relative to NPD (Figure 4D). The results identify the requirement of the immune system for a portion of the HPD-induced bone loss, and this approximate 50% reduction in HPD-induced bone loss also suggests at least 1 other mechanism may also be involved.

### Effects of dietary Pi consumption on BM and spleen T cell populations.

**Figure 5:**
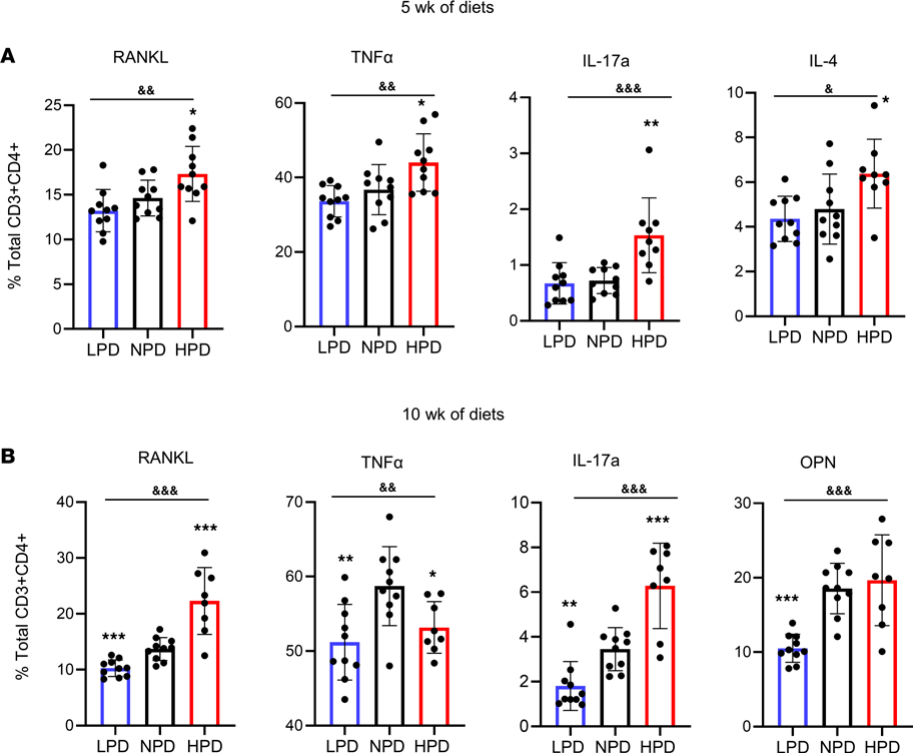
Phosphorus consumption alters inflammatory BM T cell populations. CD4^+^ T cell populations were measured from BM of the same mice described in Figure 1 by flow cytometry. (**A**) Mice on 5 weeks of diet (*n* = 10). (**B**) Mice on 10 weeks of diet (*n* = 8–10). ^&^*P* < 0.05, ^&&^*P* < 0.005, ^&&&^*P* < 0.0005 by ordinary 1-way ANOVA; *P < 0.05, **P < 0.005, ***P < 0.0005 Student’s *t* test relative to NPD. Data represent mean ± SD.

Based on these results and the previously described link between inflammatory T cells and bone loss, we sought to determine if changes in dietary phosphate consumption might alter T cell populations in the BM and/or spleen. BM and spleen T cell populations of WT mice on the different Pi diets at 5 weeks, a time of increasing separation in the bone indices between the diets, were assessed for CD3^+^CD4^+^ (CD4^+^ T cell), CD3^+^CD8^+^ (CD8^+^ T cell), CD40L^+^, CD69^+^, CD4^+^Foxp3^–^CD25^+^, CD3^+^ Naive, CD3^+^ central memory, CD3^+^ effector memory, RANKL^+^, TNF-α^+^, IFN-γ^+^, IL-4^+^, and IL-17a^+^ cell populations (Supplemental Tables 6 and 7). An analysis of percent change from total cell parent femur BM T cells of individual CD4^+^ and CD8^+^ populations identified no consistent changes in CD40L, Naive, central memory, effector memory, or Treg (CD4^+^CD25^+^FoxP3^+^) cell populations between diets, although a decrease in CD69^+^ cells and a trend (*P* = 0.07) toward a decrease in Tregs in response to the HPD was noted (Supplemental Table 6). Staining of BM CD4^+^ and CD8^+^ T cells for the inflammatory and osteoclastogenic cytokines identified a relative and absolute increase in the number of cells secreting TNF-α, RANKL, IL-4, and IL-17a in the mice fed HPD relative to NPD (Figure 5A and Supplemental Table 8). No dose-dependent, significant changes in the spleen T cell populations were detected that encompassed both percent change and absolute numbers (Supplemental Table 7). To determine if this was a chronic response, we also assessed targeted T cell populations in mice fed the various Pi diets for 10 weeks. Similar to the results obtained from mice at 5 weeks, HPD resulted in increased RANKL- and IL-17a–expressing CD4^+^ and CD8^+^ T cells compared with NPD (Figure 5B and Supplemental Table 8). Additionally, OPN-expressing T cells were quantitated, and although HPD did not increase OPN^+^ populations relative to NPD, the LPD significantly decreased CD4^+^ and CD8^+^ cell populations (Figure 5B and Supplemental Table 8). Collectively, the results provide evidence that dietary Pi influences immune cells and can lead to specific and sustained changes in effector T cell populations.

### HPD-induced bone loss can be improved by switching to a LPD.

**Figure 6:**
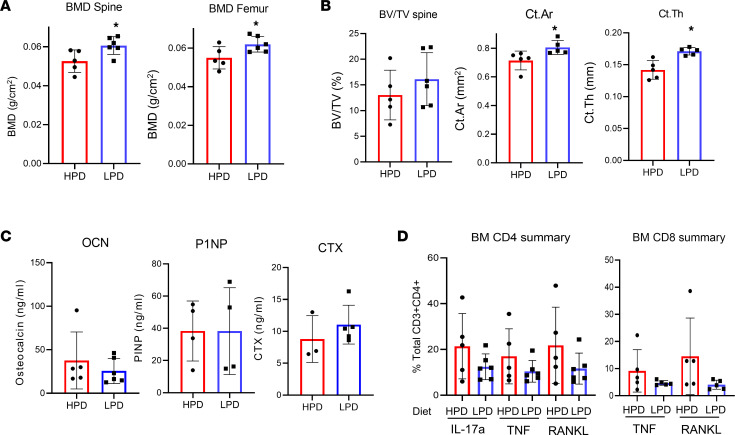
HPD-induced bone loss can be improved by switching to a low-Pi diet. Eleven C57BL/6J mice at 10 weeks of age were fed HPD for 30 weeks, at which point 6 mice were switched to LPD and 5 mice remained on HPD; all mice were sacrificed after 3 weeks. (**A**) BMD of spine and femur was measured by DXA. (**B**) μCT was used to quantitate trabecular volume of spine and cortical volume of femur. (**C**) Serum collected at sacrifice was analyzed for bone metabolism markers by ELISA. (**D**) T cell populations were measured in the BM by flow cytometry and are expressed as percentage of parent. LPD (*n* = 3 females/3 males) and HPD (*n* = 3 females/2 males). Data are shown as mean ± SD. *P < 0.05 Student’s *t* test.

The above studies describe the negative effects of a HPD and beneficial effects of LPD on bone volume in healthy mice. These studies have also detailed the sustained increase in inflammatory T cells and circulating Pi-responsive factors. To determine if initiating a LPD later in life could reverse bone loss, we fed C57BL/6J mice HPD for 20 weeks and then randomly switched half the mice to a LPD for 3 weeks (Figure 6A). Assessment of bone mineral density (BMD) by dual x-ray absorptiometry (DXA) revealed an 13% and 11% increase in BMD in the lumbar spine and femur, respectively (Figure 6A). Quantitation by μCT confirmed significant improvement in femur cortical volume in response to switching to LPD; however, spine trabecular bone was not significantly different (Figure 6B). Bone metabolism markers CTX and OCN were not strongly altered, and this possibly reflected the modest changes in trabecular bone (Figure 6C). T cell populations were measured in the BM and spleen (Supplemental Table 9) and were not found to be statistically different after only 3 weeks of switching to a LPD (Figure 6D). The results support the rapid benefit of a reduced Pi diet on bone health, whereas a more sustained change in diet may be required to benefit the immune system.

### Antibody neutralization of RANKL corrects HPD-induced bone loss.

**Figure 7:**
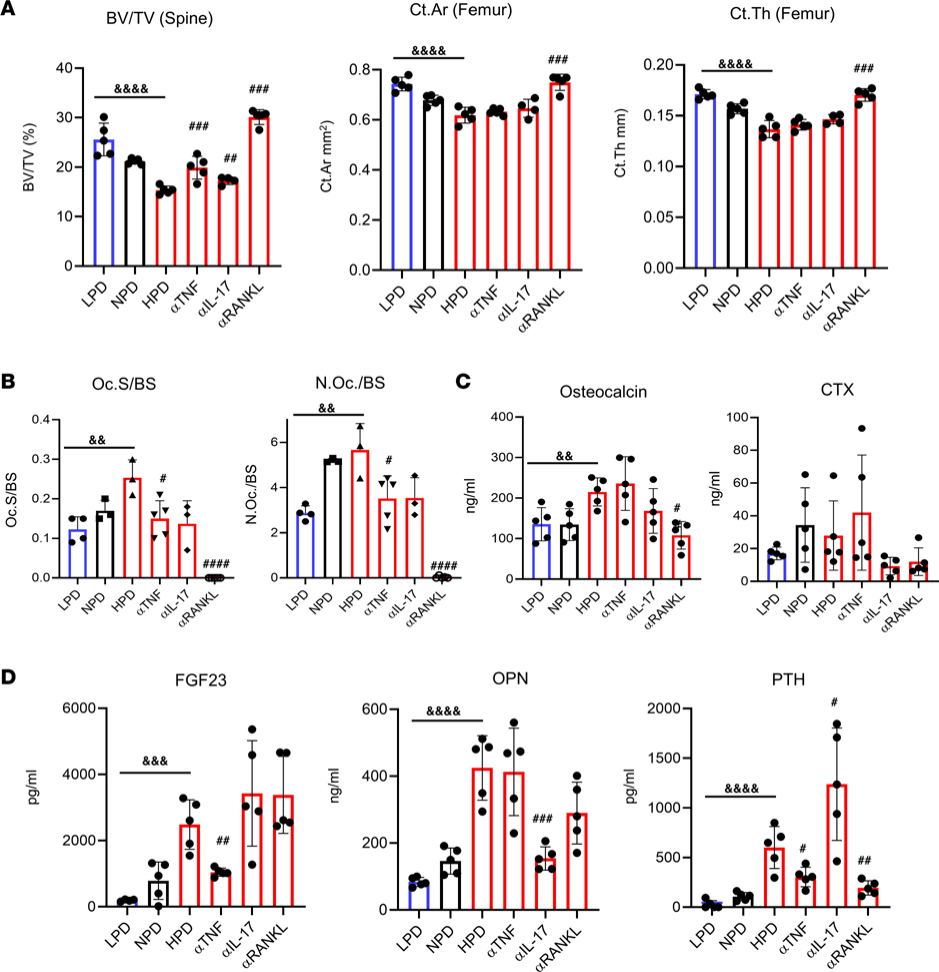
Antibody neutralization of RANKL, TNF-α, and IL-17 reduces HPD-induced bone loss. Female C57BL/6J mice (10 weeks of age) were randomized to receive LPD, NPD, or HPD and HPD with i.p. injection of control antibody or neutralizing antibodies twice weekly with isotype control (100 μg) (LPD, NPD, HPD) or targeting RANKL (100 μg; αRANKL), TNF (100 μg; αTNF), or IL-17 (200 μg; αIL-17), as indicated for 5 weeks. (*n* = 4–5/group). (**A**) Vertebral BV/TV, femoral cortical area (Ct.Ar), femoral cortical thickness (Ct.Th). (**B**) Histological sections of tibia from the same mice as in **A** were used to quantify osteoclast surface (Oc.S) per bone surface (BS) and number of osteoclasts (N.Oc) counted. (**C**) Bone metabolism markers osteocalcin and CTX were measured in serum by ELISA. (**D**) Endocrine factors FGF23, OPN, and PTH were measured in serum the mice above by ELISA. ^&^*P* < 0.05, ^&&^*P* < 0.01, ^&&&^*P* < 0.005, ^&&&&^*P* < 0.001 by 1-way ANOVA. ^#^*P* < 0.05, ^##^*P* < 0.01, ^###^*P* < 0.005, ^####^*P* < 0.01, relative to HPD. Data represent mean ± SD.

Our T cell studies identified an increase in the number of RANKL-, TNF-α–, and IL-17a–expressing T cells in response to prolonged consumption of a HPD. To determine if these cytokines are functionally involved in HPD-induced bone loss, we performed antibody-neutralization studies. Female 10-week-old C57BL/6J mice were injected i.p. with neutralizing antibodies to RANKL, TNF-α, IL-17a or control antibody twice weekly and fed HPD for 5 weeks. For comparison, mice injected with the control antibody were also fed NPD and LPD for the same time period. Assessment of vertebral trabecular and femoral cortical indices in control antibody–treated mice identified a significant reduction of bone volume in HPD-fed mice compared with NPD and an increase in bone volume when fed LPD (Figure 7A), in agreement with data presented above (Figure 1). However, HPD-fed mice injected with neutralizing antibodies targeting RANKL or TNF-α demonstrated no trabecular bone loss compared with HPD, and targeting IL-17a resulted in a small but significant inhibition of bone loss (Figure 7A). However, only targeting RANKL was sufficient to relieve the HPD-induced block in cortical gain (Figure 7A), as seen in Figure 1. Since RANKL is a key osteoclast-inducing factor, the results functionally identify the requirement of osteoclastogenesis for HPD-induced bone loss.

### Antibody neutralization of RANKL blocks HPD-driven osteoclastogenesis.

The strong inhibition of HPD-induced bone loss by neutralization of RANKL suggests that the mechanism involves increased osteoclast number and/or function. To determine if HPD increases osteoclasts, histological sections from the tibias were stained for TRAP. Quantification of osteoclast surface identified a diet-dependent increase, while antibody neutralization of TNF-α and RANKL blocked the HPD-induced increase (Figure 7B). Likewise, osteoclast number increased with increasing Pi consumption, which was again blocked by neutralization of TNF-α and RANKL (Figure 7B). Measurement of serum bone metabolism markers identified a Pi diet–induced increase in osteocalcin that was blunted with the anti-RANKL antibody and changes in CTX were not significant (Figure 7C). Analysis of serum Pi–responsive endocrine factors identified a Pi diet–induced increase in FGF23, OPN, and PTH (Figure 7D). Antibody neutralization of TNF-α, IL-17a, and RANKL had varying effects on HPD-induced levels. Targeting TNF-α decreased the HPD-induced levels of FGF23 and PTH but had no effect on OPN, whereas targeting IL-17a only decreased OPN levels. The RANKL antibody did not change HPD-induced FGF23 or OPN but strongly blunted the HPD-induced increase in PTH (Figure 7D). The results identify a dietary Pi–induced increase in osteoclasts that is inhibited by neutralization of TNF-α and RANKL and, combined with the μCT data, strongly supports the concept that HPD-induced bone loss is the result of increased osteoclast numbers and activity.

### A HPD increases RANKL expression in both BM and bone.

**Figure 8:**
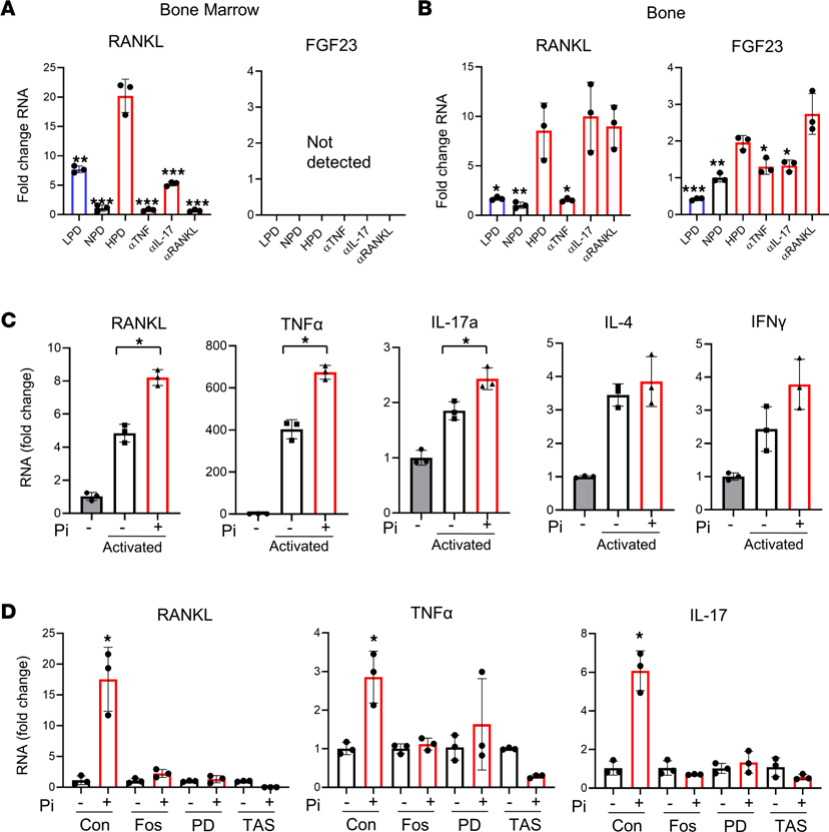
HPD and phosphate altered gene expression from bone, BM, and T cells. (**A** and **B**) RNA was isolated from BM (**A**) and bone (flushed of marrow) (**B**) from the mice described in Figure 7. qPCR of pooled cDNA from individual mice (*n* = 5) was used to quantify changes in gene expression relative to NPD as indicated. *P < 0.05, **P < 0.01, ***P < 0.005 by Student’s *t* test relative to HPD. (**C**) Primary BM pan–T cells were isolated from female 10-week-old C57BL/6J mice and either immediately harvested for RNA or treated overnight with (+) or without (–) 4 mM Pi; they were then activated for 4 hours (activated) with PMA/ionomycin. Gene expression was quantified by qPCR relative to the immediately harvested cells. Fold change was calculated relative to nonactivated, non–Pi-treated control. (**D**) Jurkat cells were treated for 48 hours with or without 4 mM Pi and in the presence of foscarnet (Fos) (1 mM), PD173074 (PD) (300 nm), or TAS-120 (50 nm); RNA was assessed by qPCR, and fold change was calculated against non–Pi-treated control for each condition. Data represent mean ± SD of samples run in triplicate. *P < 0.05 Student’s *t* test relative to control.

Although the assessment of BM T cells identified that HPD increased RANKL expression in CD4^+^ and CD8^+^ populations, osteoblast lineage cells are also known to express RANKL (*Tnfsf11*) to induce osteoclastogenesis. To better understand the source of HPD-induced RANKL, BM was flushed from femurs of the mice described in Figure 7A, and RNA was isolated from the flushed marrow and femur (bone). Analysis of gene expression by quantitative PCR (qPCR) identified a HPD-induced increase in RANKL in the BM that was inhibited by antibody targeting of RANKL, TNF-α, or IL-17a (Figure 8A). Expression of FGF23 was not detected in the BM. The RNA isolated from bone generally represents osteocyte gene expression and a HPD-induced increase in RANKL was also detected. However, unlike BM, targeting IL-17a and RANKL did not blunt the increase. Neutralizing antibodies of TNF-α strongly blunted the HPD-induced increase in RANKL expression in bone (Figure 8B). FGF23 expression was moderately increased by HPD and blunted by targeting TNF-α and IL-17a but not RANKL. OPN expression was induced by HPD, which was enhanced by the neutralization of TNF-α and blunted by targeting IL-17a. The gene expression data suggest that HPD induces RANKL RNA levels in both BM and bone cells; however, the neutralizing antibody results suggest a differential regulation between the 2 compartments.

### Elevated Pi stimulates RANKL expression in T cells.

To determine if Pi directly regulates RANKL expression in T cells, primary BM T cells were isolated from 10-week-old female C57BL/6J mice. Isolated T cells were either harvested for RNA immediately or treated with or without 4 mM Pi overnight and activated for 4 hours the next day before harvesting for RNA analysis. Primary T cells are standardly grown in RPMI medium, which contains ~5 mM Pi; therefore, 4 mM Pi was added to generally replicate the change seen in serum at the 6-hour time point (Table1). Additionally, based on previous studies, an overnight incubation with Pi is necessary to generate sustained changes in gene expression that could alter cell function. Analysis of RNA by qPCR identified an increase in Th17 cell activation cytokines, such as IL-17a, RANKL, and TNF-α but not IL-4 or IFN-γ (Figure 8C). As expected, all factors were increased by activation. Interestingly, T cells isolated from the spleen did not show a significant response to added Pi (not shown). To further investigate the potential influence of available Pi on T cells, Jurkat cells — a human T cell lymphoma line — were used. Jurkat cells were grown in RPMI medium with or without the addition of Pi for 48 hours, and RNA was harvested. Previous studies in other cell types have identified the requirement of both Pi-transport activity and FGF receptor signaling for Pi-specific responses (40); therefore, additional groups of cells were treated with the Pi-transport inhibitor foscarnet (phosphonoformic acid) as well as FGF receptor inhibitors PD173074 (FGFr1) and TAS-120 (pan-FGFr). In agreement with data from primary murine T cells, analysis of RNA by qPCR revealed that addition of Pi produced an increase in RANKL and TNF-α as well as IL-17a (Figure 8D). Furthermore, inhibition of FGFr signaling and Pi-transport activity blocked the Pi-induced increase in RANKL, TNF-α, and IL-17a. Together, the results identify the ability of elevated extracellular Pi to stimulate T cells toward a Th17 phenotype in culture, and this requires specific membrane signaling events.

## Discussion

Although a number of aging-associated health complications have correlated with either increased serum Pi or dietary Pi consumption, a causal relationship and the underlying mechanisms remain poorly defined. Furthermore, the relationship between dietary Pi consumption and serum Pi levels over time has yet to be fully elucidated. This longitudinal study on the effects of dietary Pi on bone volume, endocrine mineral metabolism factors, and immune system provides the first comprehensive assessment to our knowledge of the dynamic physiological impacts of habitually high and low dietary Pi consumption. Importantly, the HPD and LPD initially produced changes in serum Pi and Ca that were brought back into balance during the 20-week time frame of the study, yet certain health-related physiological responses remained. This result may have significant clinical implications in that an individual consistently consuming a HPD with healthy kidneys may present with unremarkable serum chemistry but may be experiencing underlying health consequences from chronically elevated Pi-responsive serum factors. Although serum Pi and Ca were eventually brought back into balance, a surprisingly sustained change in serum iron was found. An inverse relationship between dietary Pi and serum iron was first described several decades ago in humans and rats that correlated with decreased absorption and increased excretion of iron (41–46), but more recent studies have identified a decrease in serum iron in response to sustained inflammation (47). Given the general return to homeostasis of serum Pi and Ca, the reduction in serum Fe may be due to internal regulation, as opposed to absorption; however, the relationship is complex, with Fe also regulating FGF23, and this network is currently the focus of substantial research efforts.

The study identified long-term negative consequences of high dietary Pi consumption on the skeleton. Compared with NPD, a HPD accelerated trabecular bone loss over time that was sustained for the 20 weeks of the study. Although HPD did not result in substantial cortical bone loss, the gain observed with NPD over the time period did not occur in these mice. The adverse effect of excess Pi intake on bone health has been previously reported in both human and animal studies, although the underlying causes remain to be completely elucidated (26–28, 35–38). The current paradigm associated with high-phosphate diet–induced bone loss is that reduced Ca absorption leads to an increase in PTH, which stimulates osteoclastogenesis and liberates Ca from bone. However, the role of PTH in dietary Pi–altered bone metabolism remains unsettled. Early studies identified a strong temporal association between high Pi intake, increased circulating PTH, and increased bone metabolism (48), and a direct study using Ca labeling suggested that parathyroidectomy abolished bone turnover associated with a HPD in rats (49). However, more recent studies in mice have not found a requirement of PTH in the HPD bone response (50, 51). Here we identify the immune system as a previously unrecognized regulator of HPD-induced bone loss. The result is supported by an increasing number of studies demonstrating the intricate relationship between the immune system in the regulation of bone metabolism (reviewed in refs. 52, 53).

To our knowledge, this is the first report demonstrating the sustained protective effects of prolonged consumption of a reduced Pi diet on trabecular microarchitecture across time, and it emphasizes a reduced Pi diet as a potentially novel lifestyle modification in adults that might have long-term benefits for the skeleton.

LPD generated an overall increase in both trabecular and cortical bone volume. The beneficial effects of LPD on Ct.Th have been reported in a previous study in which B7D2F1 mice fed a LPD (0.3% Pi, 0.6% Ca) had thicker cortexes than those fed a NPD (0.6% Pi, 0.6% Ca) (54). Moreover, LPD-fed rats had significantly increased Ca absorption (+74%) compared with the control diet via a mechanism that likely involved reduced formation of insoluble Ca-phosphate salts in the gut (37). Increased Ca bioavailability may not fully explain the identified positive effects of a LPD on bone. These results also contribute to the growing body of literature in support of minimizing the dietary Pi burden for adults, especially as the global food supply becomes increasingly processed within the context of an aging population (55, 56).

The longitudinal study performed herein identified a robust and chronic elevation in the established Pi-responsive mineral metabolism endocrine factors FGF23 and PTH. Although it is reasonably well established that FGF23 correlates with dietary Pi consumption (57), the response of PTH and OPN are less well understood. The increase of serum PTH in response to hypocalcemia is established; however, PTH levels are known to correlate positively with Pi intake and serum Pi levels in rodents and humans (16, 18, 28, 49, 58–62). The fact that PTH also remained substantially elevated in the HPD at 20 weeks, a time when serum Pi and Ca have returned to balance and there is little difference between the diets related to bone metabolism, suggests that 1 or more additional factors influence serum PTH levels. Furthermore, PTH increased with age in mice fed NPD, as has been previously demonstrated in humans (63–67) and mice (68–70), which is suggested to be linked to aging related declines in kidney function, as seen with secondary hyperparathyroidism (71). FGF23 levels also increased with age in NPD- and LPD-fed mice, as has been previously observed (72–74), and both FGF23 and PTH correlated with a nonfasted age-related decline in Ca in the NPD-fed mice (Table 1). Taken with the data that high dietary Pi intake stimulates PTH secretion independently of age, the chance of secondary hyperparathyroidism in the elderly would be compounded in the context of a habitual HPD and could exacerbate senile osteoporosis.

OPN was also chronically elevated and represents a factor that has only been more recently identified as a Pi-responsive factor in vivo. OPN (also known as *spp1*, *Eta-1*, and *2ar*) is a secreted cytokine-like factor that can affect a range of cell types and is associated with immune response, bone metabolism, and cancer metastasis (75, 76). OPN binds to multiple receptors such as CD44 and αβ-integrins to promote changes in cell behavior such as proliferation, cell survival, cytoskeletal organization, motility, and phagocytosis. The finding of sustained elevation of serum OPN in response to consistent high Pi intake, as well as a transient decrease in response to a LPD, is potentially novel. OPN has been previously identified as directly responsive to elevated Pi in cell culture models using a variety of cell types (1, 77–79); however, the physiological meaning of this response has yet to be fully elucidated. KO of OPN in mice leads to renal and vascular calcifications (80–82), suggesting that the increase in serum OPN in response to a HPD is at least in part a defense mechanism to prevent pathological calcification. In fact, OPN has been demonstrated to physically inhibit mineralization formation through adhesion to apatite crystal surfaces (83–85).

Another unexpected finding of the study was the sustained increase in osteocalcin to high Pi consumption. Whereas CTX generally correlated with HPD-induced changes in bone over time — peaking at 5 weeks — and as observed previously (27, 86), osteocalcin seems to diverge from bone indices, suggesting a functions in the chronic Pi response beyond bone metabolism and supported by the relative lack of change in the other marker of bone formation P1NP. The HPD-driven increase in serum osteocalcin is in agreement with recent studies in both humans and animals, demonstrating a correlation between osteocalcin and Pi serum levels (27, 35, 37). In mice, osteocalcin is genomically represented by a cluster of 3 genes; *Bglap* and *Bglap2* primarily synthesized and released by mature osteoblasts during osteoid synthesis and *Bglap3* primarily expressed in kidneys and lungs (87). Interestingly, osteocalcin has previously been demonstrated to inhibit hydroxyapatite nucleation (84), suggesting the possibility that the circulating protein might act in a protective manner, to block pathological calcification, in the context of increased serum Pi.

Dysregulated Pi homeostasis has previously been linked to multiple age-related diseases, and most investigations have focused on tissue specific mechanisms. Results presented herein identify a potential common link between these pathologies in increased inflammation. Most chronic inflammatory states such as those associated with inflammatory bowel disease, chronic obstructive pulmonary disease, cystic fibrosis, and chronic kidney disease, among many others, negatively impact bone resulting in increased risk of fracture (88). T cells express many inflammatory cytokines such as TNF-α, IL-17a, and RANKL, which are also known to stimulate osteoclastogenesis and bone loss. This study identified, for the first time to our knowledge, that increasing dietary Pi consumption significantly increases BM T cell populations expressing proinflammatory cytokines TNF-α, RANKL, and IL-17a. CD4^+^IL-17a^+^ cells are defined as Th17 cells (89), and the finding that HPD increased the absolute and relative frequency of Th17 cells is particularly interesting, considering that Th17 cells are the most (if not the only) proosteoclastogenic T cell subset. Th17 cells secrete high levels of IL-17a, RANKL, and TNF-α and low levels of IFN-γ. IL-17a promotes osteoclastogenesis by stimulating the release of RANKL, TNF-α, IL-1, and IL-6 by macrophages, DCs, and stromal cells (90–92) and by upregulating RANK expression (93). TNF-α is also known to strongly affect bone metabolism through the stimulation of bone resorption and inhibition of bone formation (94, 95), and it has also been demonstrated that the upregulation of TNF-α≠producing T cells in the BM is a mechanism by which increased PTH or estrogen deficiency induces bone loss in vivo (96–98). Importantly, reducing Pi intake reduced T cells expressing RANKL, TNF-α, IL-17a, and OPN and improved bone volume, identifying a potentially novel strategy to mitigate aging-associated factors.

The study defined the functional requirement of both the immune system and, specifically, RANKL, TNF-α, and IL-17a for HPD induced bone loss. RANKL induces osteoclastogenesis through binding RANK on the surface of preosteoclasts, and the results herein identify functionally that HPD-induced bone loss requires RANKL, suggesting that bone loss is mainly occurring through increased resorption. The response is in agreement with the significant increase in bone resorption marker CTX at 5 weeks in response to HPD as well as the increase in inflammatory T cells and osteoclasts (Figure 2C, Figure 5, and Figure 7B). This study demonstrates increased RANKL^+^ T cells and the requirement of RANKL and the immune system for the negative consequences of HPD on bone, providing a potentially novel mechanism by which Pi consumption influences bone health. The results also revealed that HPD induced an increase in RANKL expression in both BM as well as bone, suggesting that — in addition to T cells — osteoblast/osteocyte lineage might also be a source. This result provides an explanation for the partial blunting of the HPD response in the RAG2 mice but complete inhibition in response to RANKL neutralization.

Whereas the RANKL neutralizing antibody blocked HPD-induced trabecular bone loss and corrected the HPD-block in cortical bone gain, the TNF-α antibody only countered trabecular loss, suggesting increased sensitivity of trabecular bone to the effects of inflammation. A number of studies have previously demonstrated the ability of anti–TNF-α agents to improve bone mass in different disease contexts (99), although an examination of trabecular verse cortical is not apparent. The lack of an effect of TNF-α neutralization on cortical bone may be due to the observation from our longitudinal study that HPD does not result in a loss of cortical bone over time but, instead, causes an inhibition of cortical gain. Interestingly, neutralization of circulating TNF-α inhibited the HPD-induced increase in circulating FGF23 and PTH as well as FGF23 gene expression in bone, in agreement with a recent translational study that found a strong correlation between plasma levels of TNF-α and FGF23 in humans (100). Additionally, inflammation has been shown to increase FGF23 expression in vitro (47, 101); the results suggest the possibility that the sustained increase in FGF23 in response to HPD reported herein might be driven in part by inflammation, and specifically TNF-α, and not entirely by serum Pi levels.

The results identified a direct response of T cells to high extracellular Pi. Both primary BM murine pan T cells as well as the human T cell line Jurkat responded to elevated Pi with increased expression of RANKL, TNF-α, and IL-17a, with less of an effect on Th1 and Th2 cell markers IL-4 and IFN-γ. The ability of Pi to stimulate changes in T cell gene expression was blocked by FGFr1 inhibition as well as the Pi-transport inhibitor phosphonoformic acid. The result is in agreement with other studies demonstrating the requirement and coordination of FGF receptors and Pi transporters for the cellular response to high extracellular Pi in kidney (102) and preosteoblast (40) cells and suggests that elevated extracellular Pi utilizes conserved signaling pathways across cell types. The physiological relevance of the T cell–based studies is supported by the agreement with HPD-induced changes in the BM in vivo. Furthermore, the result identifies that elevated extracellular Pi represents a previously unknown influence on the immune system and represents a potentially novel mechanism related to high Pi–induced bone loss.

In summary, this detailed assessment of the dynamic physiological responses to sustained HPD and LPD provides additional insight into the complex relationship between Pi availability and the skeleton. The results generate a number of important translational considerations. The demonstration that dietary Pi consumption influences T cell populations identifies a potentially physiologically meaningful link between serum Pi and the previously identified effects on pathologies associated with bone, cardiovascular, and kidney disease and cancer. In addition to known mineral metabolism markers including FGF23, PTH, and OPN, this study also identifies osteocalcin as a dietary serum Pi–responsive factor. Importantly, these factors remain elevated in the serum in response to a habitually high Pi consumption even in the context of unremarkable serum Pi and Ca. Considering the potent biological activity of these circulating factors, the sustained elevation of these factors would likely have long-term consequences. Interestingly, neutralization of TNF-α inhibited the HPD-induced increase in serum FGF23, suggesting that the sustained increase in circulating FGF23, in the absence of high serum Pi, might be in response to inflammation. The findings have important potential public health implications, given the highly processed nature of the modern food supply and the higher absorption and bioavailability of phosphorus from foods containing Pi salts. Collectively, this study provides evidence for avoiding the habitual consumption of a HPD, particularly in the aging population. Moreover, our results indicate that a reduced Pi diet may be a relatively simple yet effective strategy to maintain bone health and possibly prevent other inflammatory aging–related diseases, even in the context of normal renal function, thereby providing general long-term health benefits.

## Methods

### Study design and diets.

Female C57BL/6J mice were obtained at 8 weeks of age from The Jackson Laboratory, and RAG2^–/–^ mice were obtained from Taconic and housed in a facility with controlled conditions (temperature, 21°C–24°C; humidity, 40%–70%; light/dark cycle, 12/12 hours). At 10 weeks of age, mice were randomly assigned to varying phosphate diets (fed ad libitum) consisting of a NPD (0.6% phosphorus [TD.140507]), LPD (0.2% phosphorus [TD.110360]), or HPD (1.8% phosphorus [TD.110362]) for 0 hours (*n* = 5/diet group), 6 hours (*n* = 5) 1 week (*n* = 5), 2.5 weeks (*n* = 5), 5 weeks (*n* = 10), 10 weeks (*n* = 10), or 20 weeks (*n* = 10). For the 6-hour time point, mice were fasted overnight prior to addition of the Pi diets. For the diet-switch study, 11 C57BL/6J mice (male/female [M/F]) at 10 weeks of age were fed HPD for 30 weeks, at which point 6 mice were switched to LPD and 5 mice remained on HPD; all mice were sacrificed after 3 weeks. Study diets were designed and manufactured by Envigo to be isocaloric (3.7–3.9 kcal/g) and to keep Ca (0.6%), vitamin D (2.2 IU), and other vitamin and minerals constant. Experimental diet compositions are shown in Supplemental Table 1. Body weights were recorded on day of sacrifice. Two mice randomized to the HPD group of the 10-week time point, 2 animals in the NPD, and 3 in the LPD developed ulcerative dermatitis during the 20-week study and were not included in the analyses. No other adverse events were noted.

### Neutralizing antibody study.

Female C57BL/6J mice (The Jackson Laboratory) at 10 weeks of age were randomly assigned to receive control antibody (100 μg; clone 2A3, catalog BE0089), RANKL (100 μg; clone IK22/5, catalog BE0191), TNF-α (100 μg; clone TN3-19.12, catalog BE0244), or IL-17a (200 μg; clone 17F3, catalog BP0173) (all purchased from BioXcell) via i.p. injection twice a week. After 5 weeks on the Pi diets, mice were sacrificed, and bones, RNA, and serum were harvested for analysis.

### μCT and DXA bone imaging.

μCT was performed on the femur and third lumbar (L3) vertebrae (fixed in 70% ethanol) ex vivo to assess trabecular and cortical bone microarchitecture using a μCT40 scanner (Scanco Medical AG) that was calibrated weekly using a factory-supplied phantom. A total of 100 tomographic slices were taken from the femoral metaphysis and trabecular bone segmented from the cortical shell at a voxel size of 6 μm (70 kVp and 114 mA, and with 200 ms integration time). Cortical bone was quantified at the femoral middiaphysis from 99 tomographic slices. Projection images were reconstructed using the autocontour function for vertebral trabecular bone from approximately 350 tomographic slices. Representative samples based on mean BV/TV were reconstructed in 3D to generate visual representations. BMD analysis by DXA was performed on lumbar spine on a PIXImus2 bone densitometer (GE Medical Systems).

### Biochemical indices of serum factors.

Serum was collected at the completion of each time point under nonfasting conditions. Serum chemistry (Pi, Ca, Fe) was measured on an Alfa Wasserman Vet Axcel chemistry analyzer. Serum markers of bone metabolism and phosphate responsive endocrine factors were quantified using ELISA. All ELISA were performed according to manufacturer’s protocols and quantified by VERSAmax microplate reader (Molecular Devices). ELISA kits were purchased as follows: osteocalcin (midregion/C-terminal portion), FGF23 (C-term), FGF23 intact, and PTH (PTH1-84) (Quidel); CTX (RatLaps); P1NP (Immunodiagnostic Systems); and OPN (R&D Systems).

### Characterization of T cell populations.

Total spleen and/or BM cells were assessed for specific populations as indicated, by flow cytometry. Flow cytometry was performed on a LSR II system (BD Biosciences), and data were analyzed using FlowJo software (Tree Star Inc.). For cell-surface staining, cells were stained with anti–mouse purified CD16/32 (clone 93, catalog 101302), BV 421-TCRβ (clone H57-597, catalog 109230), APC/Cy7-CD3 (clone 17A2, catalog 100222), PerCP/Cy5.5-CD4 (clone RM4-5, catalog 100540), BV 711-CD8 (clone 53-6.7, catalog 100748), PE/Cy7-CD25 (clone PC61, catalog 102016), AF 488-CD69 (clone H1.2F3, catalog 104516), PE-CD40L (clone MR1, catalog 106506), and AF 488-CD62L (MEL-14, catalog 104420) (all from BioLegend) and PE/CF594-CD44 (clone IM7, catalog 562464) purchased from BD Biosciences). For intracellular staining, cells were incubated with leukocyte activation cocktail with BD GolgiPlug (catalog 550583, BD Biosciences) at 37°C for 4 hours. After cell fixation and permeabilization with eBioscience Intracellular Fixation & Permeabilization Buffer Set (catalog 88-8824-00, Thermo Fisher Scientific), cells were stained with anti–mouse PE-OPN (catalog IC808P) from R&D Systems, APC-TNF (clone MP6-XT22, catalog 554420) from BD Biosciences, PE-RANKL (clone IK22/5, catalog 510006), APC-IL-17A (clone TC11-18H10.1, catalog 506916), anti–mouse Alexa Fluor 488 IFN-γ (clone XMG1.2, catalog 505813), PE/Dazzle 594–IL-4 (clone 11B11, catalog 504132) (BioLegend), and APC-Foxp3 antibodies (clone FJK-16s, catalog 17-5773-82) from eBioscience were added after cell fixation and permeabilization with Intracellular Fixation & Permeabilization Buffer Set (Thermo Fisher Scientific).

### Isolation and culture of T cell populations.

Primary T cells were isolated from BM (femur, tibia, ileac crest) of 10-week-old female C57BL/6J mice (The Jackson Laboratory) using EasySep Mouse Pan-Naive T cell Isolation kit (Stemcell Technologies). Isolated T cells were cultured in RPMI (Thermo Fisher Scientific), with 10% FBS (Atlanta Biologic) and supplemented with 50 U/mL penicillin, 50 mg/mL streptomycin (Thermo Fisher Scientific). Cells were cultured overnight with or without 4 mM Pi (NaPO_4_) (MilliporeSigma; pH 7.4) as described previously (103) and then activated with a T cell stimulation cocktail (phorbol 12-myristate 13-acetate [PMA] and ionomycin, eBioscience). Jurkat cells (clone E6-1, TIB-152) were purchased from ATCC and were cultured as described for primary T cells. Cells were pretreated with inhibitors (30 minutes) and were then treated with or without 4 mM Pi followed by RNA isolation after 48 hours. Foscarnet (phosphonoformic acid) (1 mM) was purchased from MilliporeSigma and PD173074 (300 nM) and TAS-120 (50 nM) were purchased from Selleck Chemicals.

### RNA extraction, cDNA synthesis, and qPCR.

RNA was extracted using TRIzol reagent (Ambion) following the manufacturer’s protocol, cDNA was synthesized using Geneamp RNAPCR kit (Applied Biosystems), and qPCR was performed using EvaGreen qPCR master mix (Biotium) on an Bio-Rad Icycler. Primers are listed in Supplemental Table 10. Fold change was calculated using the 2^–ΔΔCt^ method (104).

### Histological sectioning and TRAP staining.

Tibias were fixed in 10% neutral buffered formalin following necropsy, decalcified in 14% EDTA (pH 7.2), dehydrated through graded ethanols, and embedded in paraffin. Resulting sections (5 μm thick) were stained with TRAP according to manufacturer’s protocol (Kamiya Biomedical Company). Analysis was performed using Bioquant Osteo software (Bioquant Image Analysis Corporation) on trabecular bone approximately 1000 μm distal to the growth plate.

### Statistics.

μCT results are expressed as mean ± SD. The baseline time-point (week 0) was excluded from statistical analyses. An ordinary 2-way ANOVA determined (a) diet effect, (b) time effect, and (c) time by diet interaction effect. When appropriate, simple effects testing followed by Tukey’s multiple-comparison test was used to determine differences across time within each diet, and different letters denote *P* < 0.05. To determine differences between LPD and HPD compared with the NPD control diet at each time point, the Dunnett’s multiple-comparison test was used. T cell populations were also assessed by ordinary 1-way ANOVA with Tukey’s multiple comparisons. Outliers were identified by ROUT (Q = 1%). Simple comparisons were performed by unpaired, 2-tailed Student’s *t* test. The data were analyzed using PRISM version 8.1.2 (GraphPad Software Inc.). All statistical tests were performed at the 5% significance level or were more stringent as indicated.

### Study approval.

Animal studies were approved by the IACUC of the Atlanta US Department of Veterans Affairs Medical Center and of Emory University (Atlanta, Georgia, USA).

## Author contributions

GRB, RP, and JLR contributed to the study design. JLR, MY, MV, JLA, and GRB performed the study and collected and analyzed data. GRB, RP, and JLR wrote, revised, and critically evaluated the manuscript.

## Supplementary Material

Supplemental data

Supplemental tables 1-10

## Figures and Tables

**Table 1:**
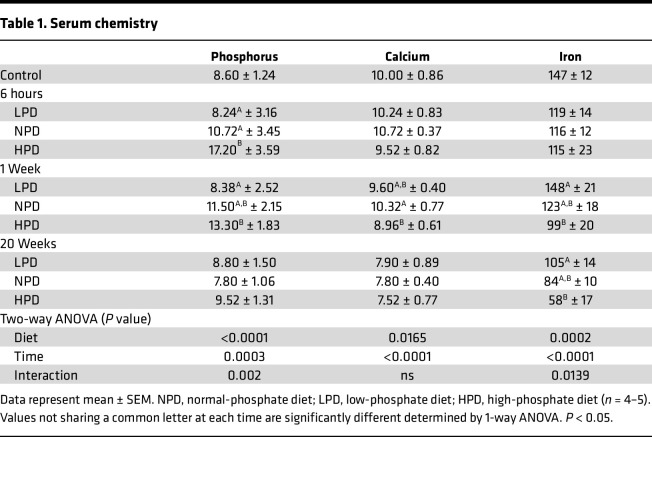
Serum chemistry

## Version 1 04/20/2023

In-Press Preview

## Version 2 05/22/2023

Electronic publication
