# Supplementary material for: Dietary phosphorus consumption alters T cell populations, cytokine production, and bone volume in mice
Source: JCI Insight. 2023 May 22;8(10):e154729. doi: 10.1172/jci.insight.154729 (PMC10322696; doi:10.1172/jci.insight.154729)
Supplement: Supplemental data [file jciinsight-8-154729-s191.pdf]

Supplemental Figure 1:

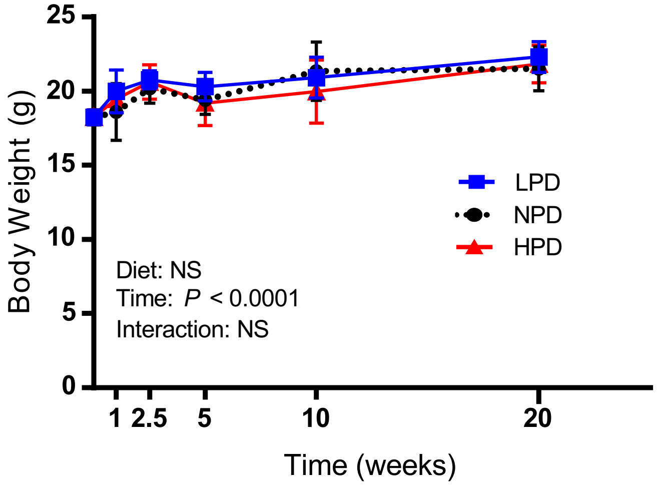

Figure 1. Weight gain did not differ between diets. 10-week old female C57BL/6J mice were randomized to receive NPD, LPD, or HPD for 1, 2.5, 5, 10, or 20 weeks and weights recorded at sacrifice. There was a significant effect of time ($P < 0.05$) on weight gain by two-way ANOVA.

Supplemental Figure 2:

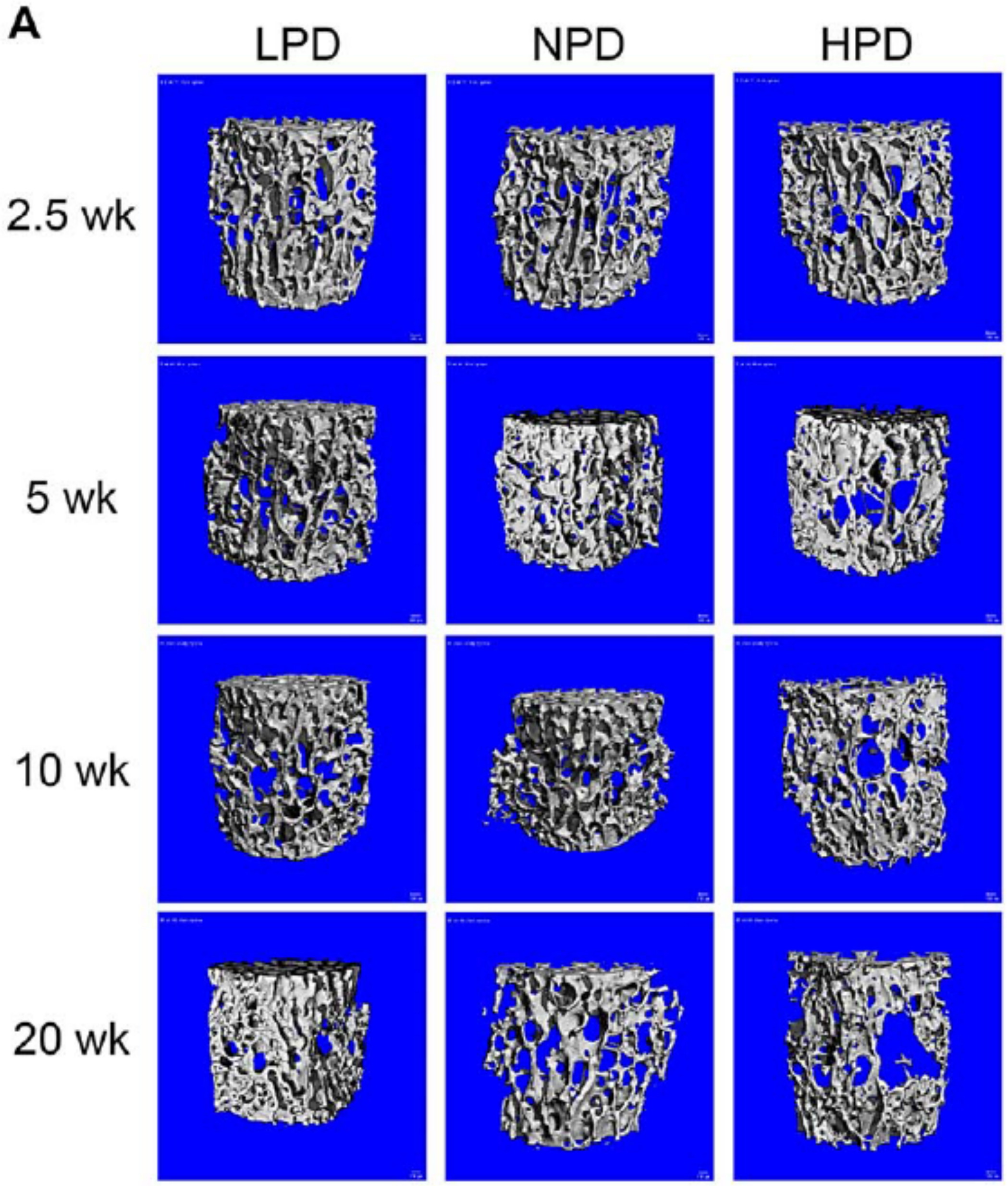

Fig. S2: Representative μCT images of L3 vertebrae from different diets at indicated time points.

Supplemental Figure 3:

Spine Trabecular indices

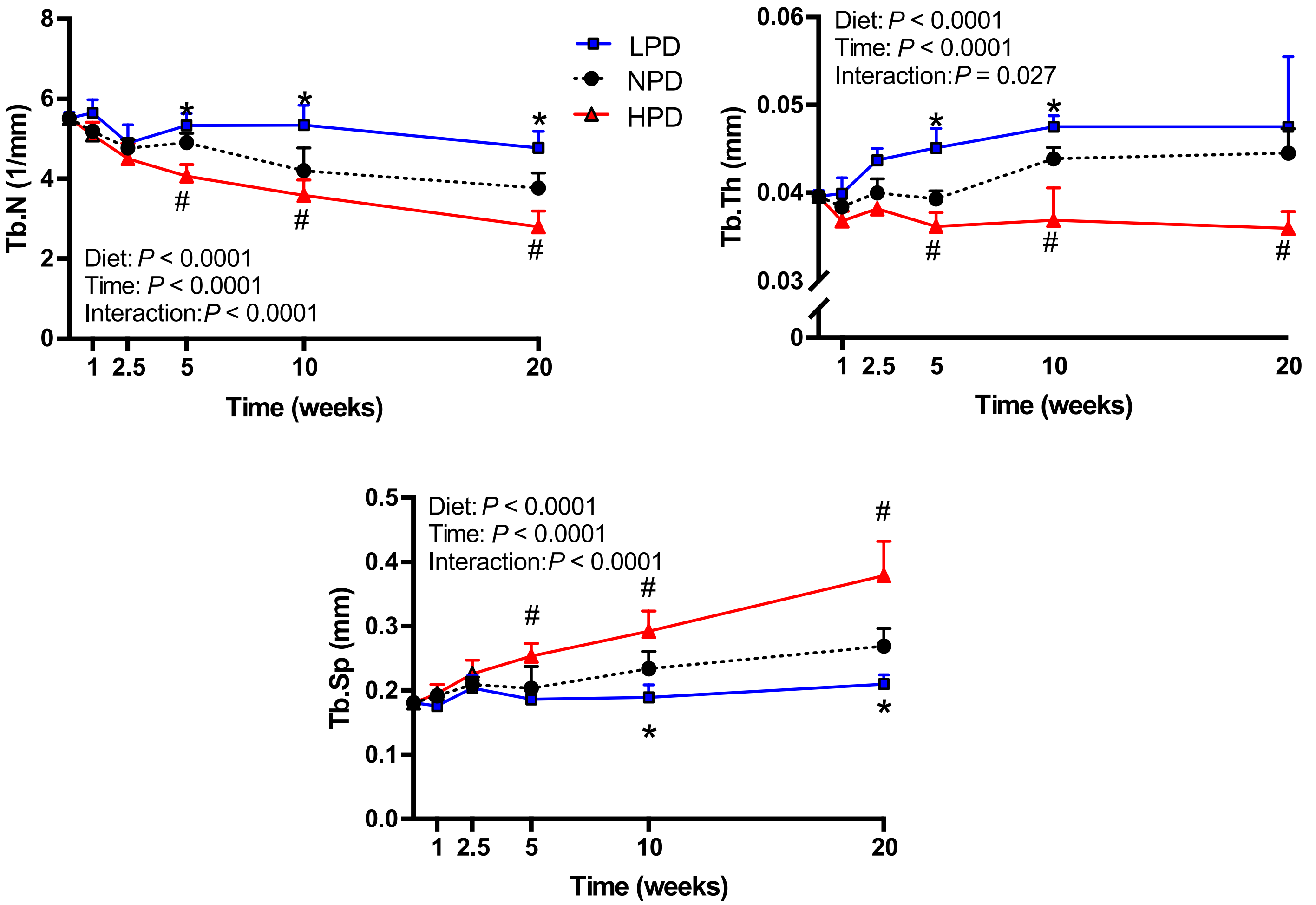

Fig.S3. Impact of phosphorus consumption on vertebral trabecular microarchitectural over time. 10-week old female C57BL/6 mice were randomized to receive NPD, LPD, or HPD for 1, 2.5, 5, 10, or 20 wks and trabecular indices quantified by µCT. Diet induced changes in vertebral Bone Volume/Tissue Volume (BV/TV) plotted against time. An ordinary two-way ANOVA determined 1) diet effect, 2) time effect, and 3) time by diet interaction effect. To determine differences between LPD and HPD compared to the NPD control diet at each timepoint the Dunnett's multiple comparisons test was used. *P<0.05 (LPD v. NPD), #P<0.05 (HPD v. NPD). Data represent mean ± SD. NPD, normal-phosphate diet; LPD, low-phosphate diet; HPD, high-phosphate diet.

Supplemental Figure 4:

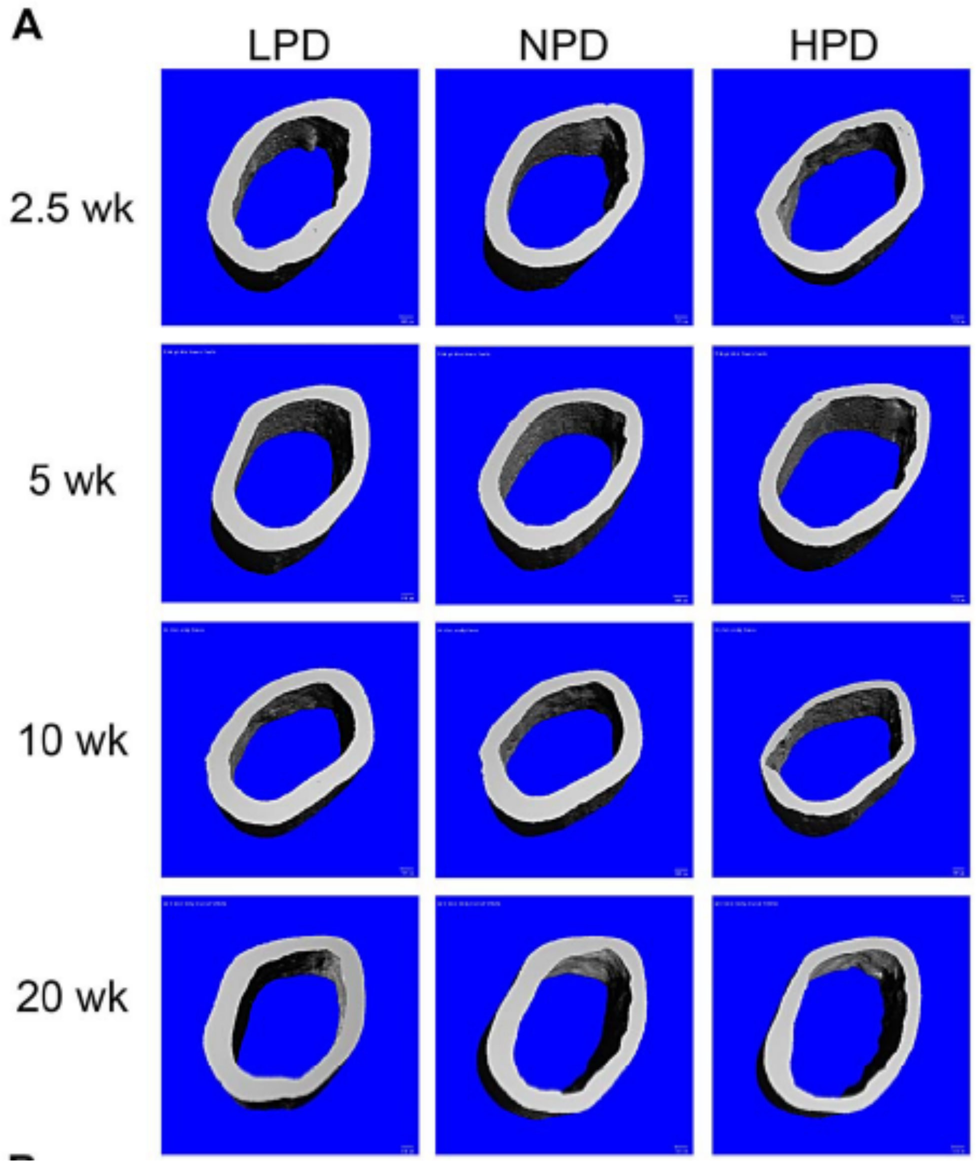

Fig. S4: Representative μCT images of femur cortical from different diets at indicated time points.

Supplemental Figure 5:

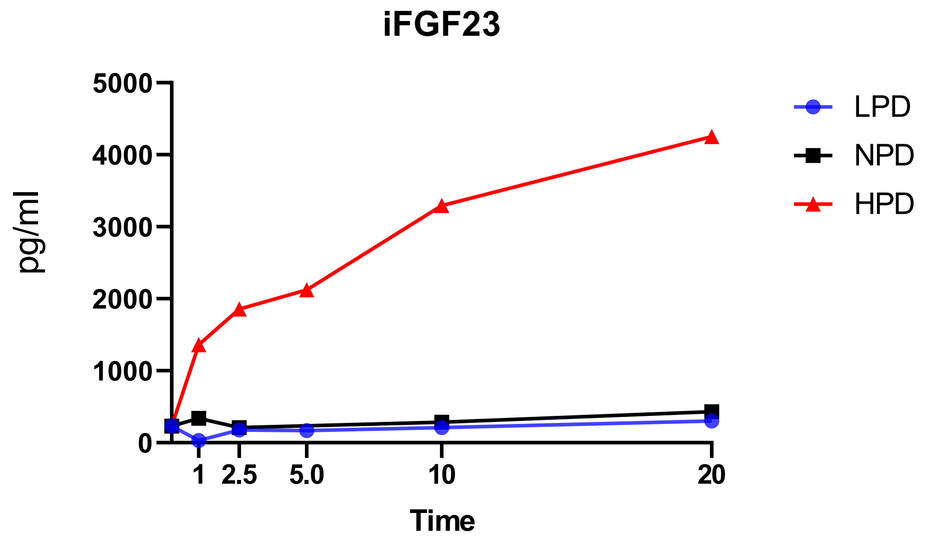

Fig. S5: Consumption of a high phosphorus diet increases intact FGF23. Serum from the mice after indicated times of diet were analyzed for intact FGF23 by ELISA. Samples for each time point were pooled from 3 mice (15ul each) and measured in duplicate and averaged.
