# Supplementary material for: Dietary phosphorus consumption alters T cell populations, cytokine production, and bone volume in mice
Source: JCI Insight. 2023 May 22;8(10):e154729. doi: 10.1172/jci.insight.154729 (PMC10322696; doi:10.1172/jci.insight.154729)
Supplement: Supplemental tables 1-10 [file jciinsight-8-154729-s192.pdf]

Supplemental Table 1. Experimental diet composition.

	LPD	NPD	HPD
Phosphate	0.2%	0.6%	1.8%
Calcium	0.6%	0.6%	0.6%
Vitamin D	2.2 IU	2.2 IU	2.2 IU
**Sources of Phosphate & Calcium (g/kg):**			
$CaCO_3$	14.9	14.9	
K_2CO_3	7.7	3.1	
KH_2PO_4	1.0	10.0	16.0
$NaH_2PO_4H_2O$	1.0	10.0	16.0
$Ca(H_2PO_4)_2H_2O$			37.5
**Sources of Sodium (g/kg):**			
NaCl	193.5	193.5	193.5
$H_{18}Na_2O_{12}Si$	3.8	3.8	3.8
$NaHCO_3$	9.2	3.7	
$NaH_2PO_4H_2O$	1.0	10.0	16.0
**Sources of Potassium (g/kg):**			
$C_6H_7K_3O_8$	593.4	593.4	593.4
K_2SO_4	121.8	121.8	121.8
K_2CO_3	7.7	3.1	
KH_2PO_4	1.0	10.0	16.0
**% kcal:**			
Kcal/g	3.9	3.8	3.7
Protein	19.3	19.4	19.9
Carbohydrate	56.9	56.6	55.4
Fat	23.8	24.0	24.6

Supplemental Table 2: Trabecular bone indices; L3 vertebrae

	Diet	Baseline	Time on Diet [weeks] 1.0	2.5	5	10	20	Percent change over time*
			Mean ± SEM					
**L3 Vertebrae**								
BV/TV [%]	LPD	22.38 ± 0.16	23.59 ± 1.06[b]	21.18 ± 1.12	24.24 ± 0.69[b]	27.52 ± 0.84[b]	24.56 ± 1.92[b]	10%
	NPD		20.38 ± 0.34[a]	18.17 ± 0.23	19.58 ± 0.43[a]	19.67 ± 0.97[a]	16.61 ± 0.61[a]	-25%
	HPD		19.26 ± 0.73[a]	17.30 ± 0.68	15.40 ± 0.38[c]	14.42 ± 1.33[c]	12.47 ± 0.56[c]	-44%
Tb.N [1/mm]	LPD	5.52 ± 0.0.6	5.65 ± 0.14[a]	4.90 ± 0.20	5.34 ± 0.09[b]	5.34 ± 0.15[b]	4.80 ± 0.17[b]	-13%
	NPD		5.19 ± 0.07[ab]	4.77 ± 0.10	4.91 ± 0.07[a]	4.20 ± 0.18[a]	3.76 ± 0.11[a]	-32%
	HPD		5.10 ± 0.14[b]	4.51 ± 0.17	4.07 ± 0.09[c]	3.59 ± 0.14[c]	2.81 ± 0.12[c]	-49%
Tb.Th [µm]	LPD	39.6 ± 0.3	39.9 ± 0.80[a]	43.7 ± 0.6[a]	45.1 ± 0.7[b]	47.5 ± 0.4[b]	48.6 ± 2[a]	20%
	NPD		38.4 ± 0.35[ab]	40.0 ± 0.7[ab]	39.0 ± 0.3[a]	43.9 ± 0.4[a]	44.0 ± 1[a]	12%
	HPD		36.8 ± 0.53[b]	38.2 ± 0.3[b]	36.2 ± 0.5[a]	36.9 ± 1.3[c]	36.0 ± 1[b]	-9%
Tb.Sp [mm]	LPD	0.18 ± 0.002	0.18 ± 0.005[a]	0.20 ± 0.009	0.19 ± 0.003[a]	0.19 ± 0.006[b]	0.21 ± 0.007[b]	16%
	NPD		0.19 ± 0.003[ab]	0.21 ± 0.005	0.20 ± 0.011[a]	0.23 ± 0.009[a]	0.27 ± 0.008[a]	48%
	HPD		0.20 ± 0.006[b]	0.23 ± 0.010	0.25 ± 0.006[b]	0.29 ± 0.011[c]	0.38 ± 0.017[c]	109%

*Computed from mean values between Baseline and 20 wk, expressed as percent change from Baseline to 20 wk
Simple effects testing (Tukey's multiple comparisons) of an effect of time within diet. Values within a row not sharing a common letter differ, P < 0.05.

Supplemental Table 3: Cortical bone indices; femur

	Diet	Baseline	Time on Diet [weeks] 1.0	2.5	5	10	20	Percent change over time*
			Mean ± SEM					
Ct.Ar [mm^2]	LPD	0.65 ± 0.01	0.78 ± 0.03	0.72 ± 0.02	0.70 ± 0.01[b]	0.77 ± 0.01[a]	0.82 ± 0.02[b]	28%
	NPD		0.75 ± 0.02	0.67 ± 0.03	0.61 ± 0.01[a]	0.73 ± 0.02[a]	0.72 ± 0.02[a]	14%
	HPD		0.74 ± 0.01	0.65 ± 0.03	0.60 ± 0.01[a]	0.62 ± 0.3[b]	0.64 ± 0.01[c]	-2%
Ct.Th [mm]	LPD	0.15 ± 0.004	0.17 ± 0.003[a]	0.17 ± 0.004[a]	0.16 ± 0.002[b]	0.18 ± 0.002[b]	0.20 ± 0.006[b]	31%
	NPD		0.17 ± 0.002[a]	0.15 ± 0.004[ab]	0.14 ± 0.003[a]	0.17 ± 0.002[a]	0.17 ± 0.004[a]	16%
	HPD		0.15 ± 0.001[b]	0.15 ± 0.007[b]	0.13 ± 0.002[c]	0.14 ± 0.007[c]	0.15 ± 0.002[c]	-4%

*Computed from mean values between Baseline and 20 wk, expressed as percent change from Baseline to 20 wk
Simple effects testing (Tukey's multiple comparisons) of an effect of time within diet. Values within a row not sharing a common letter differ, P < 0.05.

Supplemental Table 4: Serum markers of bone metabolism

	Diet	Baseline	Time on Diet [weeks] 1	2.5	5	10	20	Percent change over time*
			Mean ± SEM					
OCN [ng/ml]	LPD	116 ± 12	110 ± 14	93 ± 5[a]	112.4 ± 13[a]	28 ± 9[a]	48 ± 6[a]	-58%
	NPD		126 ± 16	97 ± 4[a]	111.7 ± 21[a]	57 ± 12[ab]	71 ± 7[a]	-39%
	HPD		131 ± 9	169 ± 7[b]	173.8 ± 23[b]	107 ± 28[b]	154 ± 3[b]	32%
P1NP [ng/ml]	LPD	108 ± 15	91 ± 17		188 ± 17		124 ± 33	15%
	NPD		64 ± 6		153 ± 21		139 ± 21	29%
	HPD		96 ± 11		206 ± 27		179 ± 29	65%
CTX [ng/ml]	LPD	27 ± 2	36 ± 8	12 ± 2	26 ± 6[a]	32 ± 10	14 ± 2	-48%
	NPD		56 ± 1	15 ± 1	34 ± 9[a]	24 ± 5	17 ± 1	-38%
	HPD		37 ± 4	36 ± 3	63 ± 17[b]	45 ± 15	20 ± 1	-26%

*Computed from mean values between Baseline and 20 wk, expressed as percent change from Baseline to 20 wk
Simple effects testing (Tukey's multiple comparisons) of an effect of time within diet. Values within a row not sharing a common letter differ, $P < 0.05$.

Supplemental Table 5: Phosphate responsive endocrine factors

	Diet	Baseline	Time on Diet [weeks] 1	2.5	5	10	20	Percent change over time*
			Mean ± SEM					
FGF23 [pg/ml]	LPD	86 ± 14	214 ± 25[a]	178 ± 7[a]	162 ± 23[a]	196 ± 34[a]	409 ± 88[a]	378%
	NPD		299 ± 44[ab]	162 ± 7[a]	325 ± 53[a]	523 ± 227[a]	645 ± 253[a]	653%
	HPD		795 ± 60[b]	1150 ± 134[b]	1610 ± 245[b]	1937 ± 428[b]	1527 ± 93[b]	1683%
OPN [ng/ml]	LPD	188 ± 15	200 ± 14[a]	140 ± 15[a]	149 ± 12[b]	303 ± 68[a]	207 ± 14[a]	10%
	NPD		257 ± 13[ab]	160 ± 5[a]	228 ± 12[a]	242 ± 12[a]	220 ± 53[a]	17%
	HPD		307 ± 11[b]	391 ± 10[b]	367 ± 20[c]	430 ± 12[b]	435 ± 32[b]	132%
PTH [pg/ml]	LPD	63 ± 10	234 ± 67	173 ± 58[a]		107 ± 65[a]	24 ± 5[b]	-63%
	NPD		295 ± 50	240 ± 99[a]		230 ± 72[a]	1035 ±287[a]	1550%
	HPD		315 ± 52	519 ± 221[a]		973 ± 343[b]	1887 ± 149[c]	2908%

*Computed from mean values between Baseline and 20 wk, expressed as percent change from Baseline to 20 wk
Simple effects testing (Tukey's multiple comparisons) of an effect of time within diet. Values within a row not sharing a common letter differ, $P < 0.05$.

Supplemental Table 6: Bone Marrow T cell Populations 5 week time point

	Percent			Absolute		
T cell Pop	LPD	NPD	HPD	LPD	NPD	HPD
Total Cells				13.7 ±0.7	14.1 ±1.4	18.9 ±0.7
CD3+	3.7 ±0.3	4.1 ±0.2	3.3 ±0.2	233850 ±19224[a]	263023 ±26367[ab]	291976 ±14603[b]
CD3+CD4+	25.8 ±1.5	26.7 ±1.2	26.9 ±1.1	59869 ±5989	71272 ±7749	77521 ±3309
CD3+CD8+	43.3 ±1.9	43.0 ±1.4	41.2 ±1.4	103817 ±11377	111961 ±10344	121391 ±8845
CD69+CD3+	20.6 ±1.0[a]	15.6 ±1.0[b]	17.4 ±0.4[ab]	34175 ±3223[a]	28376 ±2824[a]	42914 ±2.58[b]
CD69+CD3+CD4+	24.7 ±1.3[a]	19.2 ±1.8[b]	21.5 ±0.5[ab]	9144 ±838[ab]	6488 ±884[a]	13305 ±774[b]
CD69+CD3+CD8+	18.8 ±1.5[a]	15.7 ±1.2[ab]	14.4 ±0.5[b]	12282 ±1388	10356 ±989	14853 ±1001
CD4+CD40L+	22.1 ±1.0[ab]	19.4 ±1.5[a]	24.0 ±0.8[b]	8439 ±1072[a]	6974 ±1071[a]	14932 ±927[b]
CD4+Foxp3-CD25+	5.3 ±0.4	6.9 ±0.8	7.6 ±0.9	2022 ±274	2538 ±402	4827 ±747
CD4+Foxp3+	46.4 ±1.4	45.4 ±1.6	43.5 ±0.6	17011 ±1327[a]	15528 ±1484[a]	26924 ±1221[b]
CD3+Naive	22.1 ±2.1	19.7 ±1.3	20.5 ±1.8	54735 ±8453	50303 ±4711	61676 ±7408
CD3+Central Mem.	42.2 ±1.2	38.9 ±1.4	41.8 ±0.8	98506 ±7973	104089 ±13227	122677 ±7282
CD3+Effector Mem.	26.0 ±1.8	29.1 ±1.0	28.3 ±1.5	58797 ±4421	77354 ±8739	81274 ±2599
CD4+Naive	12.2 ±1.8	11.7 ±1.0	10.9 ±1.1	7778 ±1644	7821 ±856	8673 ±1124
CD4+Central Mem.	22.2 ±1.1	22.6 ±0.7	24.2 ±1.1	13229 ±1361	15876 ±1777	18913 ±1733
CD4+Effector Mem.	51.2 ±2.5	51.7 ±1.6	53.3 ±1.5	30349 ±3274[a]	37076 ±4925[ab]	41126 ±1733[b]
CD8+Naive	40.4 ±2.4	36.1 ±1.9	38.2 ±2.6	44158 ±6682	39703 ±3645	48250 ±6112
CD8+Central Mem.	40.3 ±1.1	37.8 ±1.8	41.5 ±0.9	41239 ±3899	42989 ±5110	50263 ±3551
CD8+Effector Mem.	10.2 ±1.0	12.0 ±1.0	10.4 ±0.9	9890 ±931	13920 ±2004	11953 ±428
CD3+RANKL+	7.5 ±0.52[ab]	7.0 ±0.39[a]	9.2 ±0.6[b]	15069 ±1982[a]	13716 ±1976[a]	21659 ±1397[b]
CD3+CD4+RANKL+	13.2 ±0.7[a]	14.6 ±0.6[a]	17.3 ±1.0[b]	5108 ±587	5343 ±701	9395 ±868
CD3+CD8+RANKL+	8.7 ±0.6[ab]	7.5 ±0.47[a]	10.0 ±0.6[b]	9341 ±1426[ab]	6174 ±875[a]	11037 ±717[b]
CD3+TNF+	17.0 ±0.5[a]	18.1 ±0.8[a]	24.3 ±1.2[b]	33093 ±2866[a]	36253 ±5290[a]	57740 ±3653[b]
CD3+CD4+TNF+	33.6 ±1.3[a]	36.7 ±2.1[a]	44.0 ±2.4[b]	12737 ±1252[a]	13751 ±2004[a]	23798 ±1959[b]
CD3+CD8+TNF+	13.0 ±0.8[a]	15.5 ±1.1[ab]	17.7 ±0.6[b]	13207 ±1387	13732 ±2346	19660 ±1203
CD4+IFN+	18.1 ±0.8	16.4 ±1.1	20.0 ±1.6	7606 ±929	8063 ±1149	11143 ±1369
CD4+IL-4+	4.36 ±0.3[a]	4.80 ±0.5[ab]	6.38 ±0.5[b]	1811 ±220[a]	2377 ±396[ab]	3538 ±463[b]
CD4+IL-17a+	0.67 ±0.07[a]	0.72 ±0.07[a]	1.53 ±0.22[b]	260 ±36[a]	352 ±54[a]	893 ±98[b]

Results expressed as Average ± SEM. One-way ANOVA (with Tukey's multiple comparisons) of an effect of diet.
Values within a row not sharing a common letter differ, $P < 0.05$.

Supplemental Table 7: Spleen T cell Populations 5 week time point

	Percent			Absolute		
T cell Pop	LPD	NPD	HPD	LPD	NPD	HPD
Total Cells				113 ±7.5	100 ±6.7	136 ±9.2
CD3+	28.1 ±0.9	27.6 ±0.7	28.0 ±0.6	29.6 ±2.1^{a}	25.5 ±1.6^{a}	34.7 ±2.2^{b}
CD3+CD4+	59.5 ±0.5	59.0 ±0.9	61.6 ±1.1	17.6 ±1.2^{ab}	15.1 ±0.9^{a}	21.4 ±1.4^{b}
CD3+CD8+	35.1 ±0.7	35.7 ±0.8	33.6 ±1.4	10.4 ±0.8	9.1 ±0.6	11.7 ±0.9
CD69+CD3+	8.2 ±0.38	7.8 ±0.31	7.7 ±0.30	2.25 ±.022^{ab}	1.82 ±0.12^{a}	2.46 ±0.24^{b}
CD69+CD3+CD4+	9.2 ±0.50	8.5 ±0.35	8.4 ±0.33	1.43 ±0.15	1.09 ±0.07	1.53 ±0.16
CD69+CD3+CD8+	3.5 ±0.14	3.3 ±0.12	3.4 ±0.15	0.36 ±0.03	0.30 ±0.02	0.41 ±0.03
CD4+CD40L+	1.7 ±0.10^{a}	2.5 ±0.18^{b}	1.7 ±0.14^{ab}	0.26 ±0.02	0.32 ±0.03	0.32 ±0.05
CD4+Foxp3-CD25+	1.5 ±0.14	1.5 ±0.11	1.3 ±0.06	0.23 ±0.02	0.20 ±0.02	0.24 ±0.02
CD4+Foxp3+	14.0 ±0.5^{ab}	13.3 ±0.2^{a}	14.6 ±0.3^{b}	2.15 ±0.20^{a}	1.73 ±0.11^{b}	2.64 ±0.22^{c}
CD3+Naive	59.7 ±0.9^{a}	60.1 ±1.2^{ab}	62.7 ±1.5^{b}	17.7 ±1.4^{a}	15.3 ±1.0^{a}	21.7 ±1.4^{b}
CD3+Central Mem.	17.1 ±0.6	16.2 ±0.8	16.1 ±0.6	5.0 ±0.4	4.1 ±0.3	5.2 ±0.5
CD3+Effector Mem.	16.3 ±0.6	15.7 ±0.7	14.9 ±0.9	4.8 ±0.4	4.0 ±0.3	5.2 ±0.5
CD4+Naive	58.8 ±0.9^{ab}	58.1 ±1.3^{a}	61.3 ±1.0^{b}	10.3 ±0.8^{a}	8.7 ±0.6^{b}	13.0 ±0.8^{c}
CD4+Central Mem.	10.8 ±0.4	10.5 ±0.8	11.0 ±0.4	1.9 ±0.2	1.5 ±0.1	2.3 ±0.1
CD4+Effector Mem.	22.1 ±0.9	21.7 ±0.8	20.2 ±0.9	3.9 ±0.3	3.3 ±0.3	4.4 ±0.5
CD8+Naive	68.5 ±0.9^{a}	70.7 ±0.9^{ab}	72.0 ±1.8^{b}	7.2 ±0.6^{a}	6.5 ±0.4^{a}	8.5 ±0.8^{b}
CD8+Central Mem.	22.5 ±1.0	20.6 ±0.6	20.7 ±1.2	2.3 ±0.2	1.9 ±0.1	2.4 ±0.2
CD8+Effector Mem.	4.2 ±0.4	3.6 ±0.2	3.3 ±0.3	0.44 ±0.06	0.33 ±0.03	0.37 ±0.04
CD3+RANKL+	9.95 ±0.9^{ab}	7.97 ±0.4^{a}	11.9 ±0.8^{b}	2.85 ±0.3^{ab}	1.95 ±0.2^{a}	3.78 ±0.4^{b}
CD3+CD4+RANKL+	17.4 ±1.2^{ab}	14.6 ±0.6^{a}	20.0 ±1.1^{b}	2.76 ±0.3^{ab}	1.95 ±0.2^{a}	3.58 ±0.4^{b}
CD3+CD8+RANKL+	8.2 ±0.9^{a}	5.9 ±0.3^{b}	9.9 ±0.7^{ac}	0.93 ±0.11 ^{a}	0.59 ±0.05^{b}	1.23 ±0.09^{ac}
CD3+TNF+	48.9 ±1.8	48.1 ±1.2	48.1 ±1.5	13.9 ±1.1^{ab}	11.6 ±0.8^{a}	15.3 ±1.1^{b}
CD3+CD4+TNF+	60.4 ±1.6	60.7 ±0.7	61.2 ±1.6	9.5 ±0.7^{ab}	8.0 ±0.6^{a}	10.9 ±0.8^{b}
CD3+CD8+TNF+	32.5 ±2.0	30.8 ±1.9	29.2 ±1.9	3.72 ±0.35	3.01 ±0.21	3.66 ±0.32
CD4+IFN+	5.8 ±0.5	5.5 ±0.4	5.5 ±0.6	0.838 ±0.094^{ab}	0.659 ±0.079^{a}	0.992 ±0.152^{b}
CD4+IL-4+	1.7 ±0.2	1.6 ±0.1	2.0 ±0.2	0.253 ±0.031	0.193 ±0.018	0.343 ±0.048
CD4+IL-17a+	0.19 ±0.02	0.30 ±0.03	0.25 ±0.03	0.027 ±0.003	0.034 ±0.003	0.042 ±0.005

Results expressed as Average ± SEM. One-way ANOVA (with Tukey's multiple comparisons) of an effect of diet.
Values within a row not sharing a common letter differ, $P < 0.05$.

Supplemental Table 8: Bone Marrow T cell Populations 10 week time point

	Frequency of Parent			Absolute		
T cell Pop	LPD	NPD	HPD	LPD	NPD	HPD
Total cells (10^6)				12.1 ±0.7^{a}	19.8 ±1.1^{b}	19.0 ±2.5^{b}
Total TcrB+ cells	1.9 ±0.1	2.7 ±0.5	2.7 ±0.3	216740 ±16607^{a}	482314 ±73841^{b}	522881 ±46880^{b}
CD4+	35.1 ±2.4	29.6 ±2.7	29.3 ±3.5	78232 ±10227	158859 ±41320	164106 ±29638
CD8+	33.1 ±2.0^{a}	35.2 ±1.1^{ab}	39.3 ±1.0^{b}	72225 ±7102^{a}	170764 ±27436^{b}	204163 ±17485^{b}
Total-OPN+	16.6 ±0.7^{a}	21.9 ±1.0^{b}	22.4 ±1.6^{b}	36050 ±3398^{a}	101010 ±11176^{b}	113846 ±26398^{b}
CD4+OPN+	10.5 ±0.6^{a}	18.6 ±1.1^{b}	19.7 ±2.2^{b}	8478 ±1437^{a}	27546 ±5783^{b}	29393 ±5131^{b}
CD8+OPN+	15.0 ±0.7^{a}	21.6 ±1.2^{b}	22.2 ±1.6^{b}	10975 ±1322^{a}	36557 ±5446^{b}	45670 ±5443^{b}
Total-RANKL+	21.8 ±1.0^{a}	29.5 ±1.1^{b}	39.4 ±2.4^{c}	46445 ±3117^{a}	137819 ±16872^{b}	202357 ±17946^{c}
CD4+RANKL+	10.3 ±0.5^{a}	13.7 ±0.6^{a}	22.3 ±2.1^{b}	8192 ±1246^{a}	17295 ±5995^{ab}	30851 ±8840^{b}
CD8+RANKL+	23.1 ±0.8^{a}	32.4 ±1.5^{b}	41.6 ±2.2^{c}	16652 ±1636^{a}	54874 ±8542^{b}	84311 ±7642^{c}
Total TNF+	37.5 ±1.2^{a}	44.9 ±1.2^{a}	41.1 ±6.0^{ab}	121026 ±15125^{a}	307229 ±59185^{b}	305730 ±47010^{b}
CD4+TNF+	51.2 ±5.1^{a}	58.7 ±5.3^{b}	53.2 ±3.5^{ab}	51076 ±9967	126363 ±42897	133408 ±28502
CD8+TNF+	35.7 ±5.3^{a}	43.7 ±5.4^{b}	43.3 ±3.8^{b}	42887 ±4433^{a}	120240 ±24008^{b}	122386 ±23753^{b}
CD4+IL17+	1.8 ±1.1^{a}	3.5 ±1.0^{b}	6.3 ±1.9^{c}	1615 ±281^{a}	6252 ±1557^{a}	15691 ±4316^{b}

Results expressed as Average ± SEM. One-way ANOVA (with Tukey's multiple comparisons) of an effect of diet.
Values within a row not sharing a common letter differ, $P < 0.05$.

Supplemental Table 9: BM and spleen T cell Populations 33 week time point

	Bone Marrow			Spleen	
T cell Pop (%)	LPD	HPD		LPD	HPD
CD4+IL-17A+	12.4 ±2.3	21.4 ±6.4		2.32 ±0.25	2.34 ±0.23
CD4+TNF+	10.5 ±1.9	17.0 ±5.4		3.88 ±0.25	4.03 ±0.37
CD4+RANKL+	11.6 ±2.8	21.8 ±7.4		3.27 ±0.31	3.81 ±0.51
CD8+TNF+	6.98 ±2.30	9.17 ±3.50		2.93 ±0.20*	3.78 ±0.20
CD8+RANKL+	4.06 ±0.75	8.55 ±2.45		1.35 ±0.11	3.83 ±1.36
CD25+	13.9 ±1.84	19.2 ±3.60		8.08 ±1.47	11.4 ±1.82
CD69+	25.6 ±2.15	23.9 ±3.75		10.9 ±1.48	13.3 ±1.67
Naive	2.60 ±0.48	2.37 ±1.09		37.8 ±3.9	33.7 ±6.4
Central Memory	14.0 ±2.02	12.3 ±2.67		19.3 ±1.1	16.9 ±0.7
Effector Memory	69.9 ±2.14	69.6 ±3.48		36.5 ±3.8	40.9 ±5.6

Results expressed as Average ± SEM. *P < 0.05 by student's t test.

Supplemental Table 10: Primers used for qRT-PCR

Gene	Forward	Reverse	Source
**Mouse**			
UBC	TCCAGAAAGAGTCCACCCTG	GACGTCCAAGGTGATGGTCT	1
FGF23	TTTCCCAGGTTCGTCTAGG	CTCGCAGGTGACTCTCAG	2
IL-17a	TCAGCGTGTCCAAACACTGAG	CGCCAAGGGAGTTAAAGACTT	3
RANKL	GACTCCATGAAAACGCAGAT	GAAAGGCTTGTTTCATCCTCC	1
TNFα	GCCTCTTCTCATTCCTGCTT	CACTTGGTGGTTTGCTACGA	4
Il-4	GGTCTCAACCCCCAGCTAGT	GCCGATGATCTCTCTCAAGTGAT	1
IFNγ	GCCACGGCACAGTCATTGA	TGCTGATGGCCTGATTGTCTT	1
**Human**			
18S	CAGCCACCCGAGATTGAGCA	TAGTAGCGACGGGCGGTGTG	1
IL-17a	TCCCACGAAATCCAGGATGC	GGATGTTCAGGTTGACCATCAC	3
TNFα	GAGGCCAAGCCCTGGTATG	CGGGCCGATTGATCTCAGC	1
RANKL	CAACATATCGTTGGATCACAGCA	GACAGACTCACTTTATGGGAACC	1

RANKL:(*Tnfsf11*). Primers were synthesized by Integrated DNA Technologies, Inc. (Coralville, IA)

1. Design by PrimerBank (Wang X, and Seed B. A PCR primer bank for quantitative gene expression analysis. *Nucleic Acids Res.* 2003;31(24):e154. And. Wang X, Spandidos A, Wang H, and Seed B. PrimerBank: a PCR primer database for quantitative gene expression analysis, 2012 update. *Nucleic Acids Res.* 2012;40(Database issue):D1144-9).
2. Stubbs JR, He N, Idiculla A, Gillihan R, Liu S, David V, et al. Longitudinal evaluation of FGF23 changes and mineral metabolism abnormalities in a mouse model of chronic kidney disease. *J Bone Miner Res.* 2012;27(1):38-46.
3. Li JY, D'Amelio P, Robinson J, Walker LD, Vaccaro C, Luo T, et al. IL-17A Is Increased in Humans with Primary Hyperparathyroidism and Mediates PTH-Induced Bone Loss in Mice. *Cell Metab.* 2015;22(5):799-810.
4. Yang YH, Morand EF, Getting SJ, Paul-Clark M, Liu DL, Yona S, et al. Modulation of inflammation and response to dexamethasone by Annexin 1 in antigen-induced arthritis. *Arthritis Rheum.* 2004;50(3):976-84.
